# The Legal Vulnerability Model for Same-Sex Parent Families: A Mixed Methods Systematic Review and Theoretical Integration

**DOI:** 10.3389/fpsyg.2021.644258

**Published:** 2021-03-16

**Authors:** Magdalena Siegel, Constanze Assenmacher, Nathalie Meuwly, Martina Zemp

**Affiliations:** ^1^Department of Developmental and Educational Psychology, University of Vienna, Vienna, Austria; ^2^Department of Public Health, Institute of Tropical Medicine, Antwerp, Belgium; ^3^Department of Clinical and Health Psychology, University of Vienna, Vienna, Austria; ^4^Department of Psychology, Institute for Family Research and Counseling, University of Fribourg, Fribourg, Switzerland

**Keywords:** legal vulnerability, sexual orientation law, structural stigma, same-sex families, minority stress, sexual minorities, systematic review, same-gender families

## Abstract

Globally, parents and children in same-sex parent families are impacted by many laws related to the parental sexual orientation. These laws vary considerably from one country to another, ranging from full legal recognition to criminalization. The psychological consequences of living in an ambiguous or hostile legal climate likely interfere with parental health, family functioning, and child development. However, a systematic evidence synthesis of the pertinent literature and its placement within a broader psychological model are currently lacking. The aims of this review were thus (1) to systematically review qualitative and quantitative evidence on the impact of sexual orientation laws on same-sex parent families in key domains and (2) to place these findings within a broader model informed by minority stress and family theories. Our review was preregistered and conducted in line with PRISMA guidelines. We searched for qualitative, quantitative, and mixed methods studies on the impact of sexual orientation laws on target outcomes (parental health, family functioning, child outcomes) via systematic database search (PubMed, Scopus, Web of Science) and forward-backward searches. Fifty-five studies published between 1999 and 2020 were eligible for inclusion and were synthesized using a data-based convergent synthesis design. Thirteen descriptive and three overarching analytical themes were identified through thematic synthesis. Linking our findings with minority stress and family theories, we propose a novel legal vulnerability model for same-sex parent families. The model posits that legal vulnerability constitutes an increased risk for parental and child health as well as family functioning through individual and shared pathways between family members. Additionally, the model introduces counteractions that families engage in on the personal, familial, and systemic level to mitigate the impact of legal vulnerability, as well as moderators on the contextual, familial, couple, and individual level that modulate this impact. Implications for research and clinical practice are discussed.

## Introduction

Legal recognition and freedom from persecution have long been recognized as pivotal to the well-being and functioning of same-sex parent families by social scientists (Herek, 2006; Patterson and Farr, 2016), professional associations (e.g., American Psychological Association, 2011, 2020a,b; Manning et al., 2014; American Medical Association, 2015), and supranational organizations alike (e.g., UNICEF, 2014; European Commission, 2020). As these position statements show, *whether* same-sex parent families should be granted equal rights is not a question in need of scientific inquiry. However, much less is known about *how* access to equal rights (or the lack thereof) impacts both parents' and children's health in these families (Moore and Stambolis-Ruhstorfer, 2013; Umberson et al., 2015), representing an important lacuna in family theory and an overlooked component in clinical practice.

This lack of formalization in studying the impact of structural factors such as laws on same-sex parent families is not surprising: Research on same-sex parent families in general has been criticized as lacking explicit theoretical frameworks and integration within the broader family psychology literature (Farr et al., 2017; van Eeden-Moorefield et al., 2018), while scientific inquiry into structural determinants of sexual minority health is a recent phenomenon in itself (e.g., Hatzenbuehler, 2010, 2016).

The purpose of this review is to address both points by (i) systematically summarizing the pertinent evidence on the impact of laws and legal recognition on the health and family functioning of same-sex parent families, (ii) placing it within existing minority stress and family theories, and (iii) outlining implications for research and practice. We do so by introducing the concept of legal vulnerability, which – as we theorize in a novel integrative model – links the impact of laws and legal recognition of same-sex parent families with individual and family-related outcomes.

### The Legal Landscape for Same-Sex Parent Families

Globally, the legal landscape for sexual minorities is varied and in constant flux (Waaldijk et al., 2017; ILGA World et al., 2020): In 2020, sexual minorities could face the death penalty (11 countries) or imprisonment (57 countries) in some parts of the world, while enjoying access to civil marriage (28 countries) in others (ILGA World et al., 2020). A multitude of laws (collectively referred to as sexual orientation laws) regulate the lives of sexual minorities in many other areas as well, including protection from hate crimes or discrimination, freedom of assembly, or even blood donation (ILGA World et al., 2020).

For same-sex parent families, laws related to the recognition or criminalization of the family or its members are particularly salient^1^. The legal recognition of same-sex parent families refers to (i) the recognition of the interparental relationship through civil union or marriage, as well as (ii) the recognition of the parent-child relationship through adoption (Shapiro, 2020). Adoption laws include the right to jointly adopt a biologically unrelated child (by both parents) and the right to adopt a partner's child (i.e., second-parent adoption). The combination of marriage and adoption laws can create varied and insufficient legal ties between family members, as some countries legally recognize the interparental but not the parent-child-relationship (or vice versa), require marriage in order to adopt, or lack automatic co-parent recognition at childbirth (see e.g., ILGA-Europe, 2020; ILGA World et al., 2020).

The tangible benefits of legal ties between family members are numerous (Shapiro, 2020). A legally recognized interparental relationship is associated with important financial and material benefits and safeguards, including fiscal relief, insurance, and inheritance. A legally recognized parent-child relationship ensures the child's access to these and other important resources (e.g., alimony). Additionally, non-legal parents lack power of attorney for their child in educational and healthcare contexts, which may prevent them from signing school documents or accompanying their child to medical visits (Pawelski et al., 2006; Shapiro, 2020).

Sexual orientation laws can also serve to criminalize sexual minorities (and thus, same-sex parents), for example through the criminalization of the parental sexual orientation (most commonly by penalizing same-sex sexual behavior; ILGA World et al., 2020), or through so-called “propaganda laws.” These laws penalize the “promotion” of non-traditional sexual relations toward minors, thereby constituting a source of stress and anxiety for same-sex parent families in particular (Zhabenko, 2019) and legitimizing discrimination and stigma among the public (Hylton et al., 2017).

In recent years, many countries—particularly European and North American—have seen an unprecedented shift in the legal recognition of same-sex parent families (Waaldijk, 2020) and in concurrent attitudes of the general public (Baunach, 2012; Smith et al., 2014). Still, same-sex parent families do not enjoy equal rights in most of these countries. For example, in an overview of sexual orientation legislation in 49 European countries (www.rainbow-europe.org), only two (Belgium and Malta) are listed as providing full equality in the category “Family” in 2020.

Globally, the noticeable legal progress in some parts of the world stands in stark contrast to its halt or even regress in many others. It is estimated that the majority of sexual minorities worldwide conceal their sexual orientation (Pachankis and Bränström, 2019). The number of countries where a non-heterosexual orientation is illegal (35%) currently exceeds the number of countries that recognize the interparental (same-sex marriage legal in 14%, civil union in 18%) or the parent-child relationship (joint-adoption legal in 14%, second-parent adoption in 16%; ILGA World et al., 2020).

### Sexual Orientation Laws and Individual Sexual Minority Health

The detrimental impact of restrictive sexual orientation laws (e.g., constitutional marriage bans), lacking legal relationship recognition or protection from discrimination, and a country's overall (socio-)legal climate have been repeatedly linked to adverse physical and mental health outcomes in sexual minority youth and adults. These include reduced life satisfaction (Pachankis and Bränström, 2018), impaired physical health (Kail et al., 2015), increased general mental distress (Rostosky et al., 2009; Tatum, 2017; Hatzenbuehler et al., 2018; Raifman et al., 2018), increased psychiatric morbidities (Hatzenbuehler et al., 2009, 2010; Everett et al., 2016), and suicide attempts (Raifman et al., 2017). Studies from countries with criminalizing legislation, such as Russia (Hylton et al., 2017), Nigeria (Schwartz et al., 2015), Senegal (Poteat et al., 2011), and India (Rao and Mason, 2018; Rao et al., 2020), document the pervasive fear, stigma, and negative mental and physical health sequelae among sexual minorities due to their illegal sexual orientation. Notably, it is not only the impact of adverse legislation that has been found to be detrimental to sexual minority health, but also campaigns and hateful rhetoric surrounding them (e.g., before a referendum; Russell and Richards, 2003; Maisel and Fingerhut, 2011; Frost and Fingerhut, 2016; Flores et al., 2018).

Sexual orientation laws have also been found to target stressors specific to sexual minority populations. These stressors are collectively termed minority stress (Meyer, 2003) and pose additional sources of stress on the distal (e.g., through discrimination and prejudicial events) and proximal level (e.g., through concealment of the sexual orientation, internalized homonegativity, and expectations of and sensitivity to rejection). Specifically, sexual orientation laws and concomitant societal attitudes have been linked to discrimination, victimization and bullying (Everett et al., 2016; Hatzenbuehler et al., 2018, 2019), sexual orientation concealment (Pachankis et al., 2015; Charlton et al., 2016; Riggle et al., 2017; Pachankis and Bränström, 2018), rejection sensitivity (Pachankis et al., 2014), and internalized homonegativity (Berg et al., 2013).

### Sexual Orientation Laws and Family Functioning

On the family level, lacking legal recognition of family relationships places an economic burden by the need to secure a legally binding family structure by means of wills and power of attorney (e.g., Perrin et al., 2013). Psychologically, being in a legally unrecognized family has been found to be a chronic source of stress, anxiety, and safety concerns for both parents (e.g., Park et al., 2016; Zhabenko, 2019) and children (Goldberg and Kuvalanka, 2012; Goldberg et al., 2013). Conversely, the legalization of marriage or the granting of adoption rights have been found to foster family stability and security (e.g., Surtees, 2011; Rawsthorne, 2013). The legal recognition of family relationships (or the lack of it, respectively), decreases (or induces) doubts about being a legitimate parent (e.g., Padavic and Butterfield, 2011; Bacchus, 2018), and impacts interparental (Butterfield and Padavic, 2014), parent-child (Kazyak, 2015; Gash and Raiskin, 2018; Malmquist et al., 2020), and sibling relationships (Goldberg and Allen, 2013; Malmquist et al., 2020).

### Objectives

Several key areas of parent and child health and family functioning seem to be affected by the legal climate and recognition of family relationships. However, the findings outlined above are characterized by considerable heterogeneity in terms of contexts, populations, study designs, and theoretical underpinnings and lack a unifying framework.

Accordingly, our review has two goals: First, we systematically review qualitative and quantitative evidence on the impact of sexual orientation laws on same-sex parent families on the following domains: (a) parental and child health, (b) family relationships and functioning (i.e., relationship quality, conflict, parenting), and (c) child educational and cognitive outcomes.

Second, we aim at deriving an integrative model that elucidates possible pathways through which sexual orientation laws affect same-sex parent families. For this purpose, we place our findings within well-established theories and key literature pertaining to minority stress (Meyer, 2003; Hatzenbuehler, 2009; LeBlanc et al., 2015; Feinstein, 2020), family resilience (Walsh, 2016), and parenting models (Feinberg, 2003).

## Methods

### Protocol, Adherence to Review Guidelines, and Eligibility Criteria

The protocol for this review was prepared according to PRISMA-P guidelines (Shamseer et al., 2015) and preregistered on May 13, 2020 (https://osf.io/efgkr/). Eligibility criteria (see Table 1 and the study protocol), information sources, search strings, and data collection and synthesis methods were specified in advance. The review was conducted in line with PRISMA guidelines (Moher et al., 2009; see OSF-Supplement S1 for the PRISMA-checklist) and guided by the ENTREQ statement for qualitative research synthesis (Tong et al., 2012).

**Table 1:** Inclusion and exclusion criteria according to population, intervention/exposure, controls, outcome, study type (PICOS).

**Criterion**	**Inclusion**	**Exclusion**
Publication and study type	• Peer-reviewed articles, book chapters, dissertation theses, unpublished research reports • Empirical qualitative, quantitative, or mixed methods studies • Meta-analyses if legal variation was established at the between-study level	• Books, master theses • Non-empirical works (e.g., letters to the editor, position papers, conceptual contributions) • Narrative reviews
Population	• Members of a same-sex parent family^a^ (either as a parent or as a child^b^)	• Planned collaborative coparenting arrangements with more than two parents • Non-heterosexual single parents by choice • Mixed samples of relatives or friends of same-sex parents • Non-heterosexual parents in mixed-sex relationships • Sexual minority youth (unless growing up in a same-sex parent family) • Same-sex couples with unclear parental status or without children
Intervention/exposure	• Operationalization (quantitative study) or discussion (qualitative study) of one or more sexual orientation laws^c^ (see Siegel et al., 2019) or the legal climate for same-sex parent families	• Laws related to asylum and sexual orientation, military laws and sexual orientation, local policies (e.g., at the workplace), laws concerned with gender identity (e.g., recognition of trans parenthood), laws related to sexuality in general (e.g., sex work) • Other influences at the structural level (e.g., societal attitudes toward sexual minorities)
Controls	–	–
Outcome	• Operationalization (quantitative study) or discussion (qualitative study) of one of the following outcomes: • Mental or physical health of parents or children (including general and minority-specific health-related protective and risk factors) • Family relations and family functioning (e.g., relationship quality, interparental or parent-child conflict, parenting) • Cognitive outcomes and educational attainment (child only)	• Material/financial outcomes, even if health-related (e.g., access to health insurance) • Parenthood aspirations • Family formation

*Detailed information and examples regarding specific criteria are outlined in the study protocol (https://osf.io/efgkr/)*.

a *A romantic relationship between the parents at time of data collection was not an inclusion criterion (i.e., parents could have been separated at time of data collection)*.

b *No age limit (e.g., < 18 years) was set for the child generation*.

c *Although not specified in the study protocol, this also includes studies that investigate the impact of having to use different legal means than mixed-sex parent families, e.g., second-parent adoption by the non-birth mother after the birth of a child conceived via donor insemination*.

### Information Sources and Search Strategy

We searched three electronic databases (*PubMed, Scopus, Web of Science*) through May 10, 2020 using multiple combinations of search terms based on free and controlled vocabulary (100+ individual terms, 14 sets) related to (a) sexual minorities and (b) sexual orientation laws (search strings for all databases are provided under https://osf.io/hnp8g/). The systematic literature search was conducted by the first author as part of an on-going systematic review on the impact of sexual orientation laws on sexual minorities (Siegel et al., 2019). For the purpose of this review, records retrieved by this search were filtered by the following terms in titles, abstracts, or keywords: parent^*^, mother, father, couple, child^*^, offspring, adolesc^*^, teen^*^, youth, family, families, familial (asterisks indicate wildcards). Notably, the review by Siegel et al. (2019) addresses the impact of sexual orientation laws on individuals (not the family unit); thus, there is no overlap between reviews.

We did not systematically search gray literature databases due to the complexity of our search string and the limited possibilities of these databases to handle complex Boolean combinations and truncations (see e.g., Gusenbauer and Haddaway, 2020). Instead, we conducted forward (*Google Scholar*) and backward searches of (i) studies eligible for inclusion retrieved via our database search, as well as (ii) unsystematic reviews on same-sex parenting (Biblarz and Savci, 2010; Reczek, 2020) and associated legal vulnerabilities (Kazyak and Woodell, 2016; Kazyak et al., 2018). We deemed this approach reasonable given the extensive coverage of *Google Scholar* (including thesis and other gray literature databases) as well as the comprehensiveness of our database search.

### Study Selection

**Figure 1:**
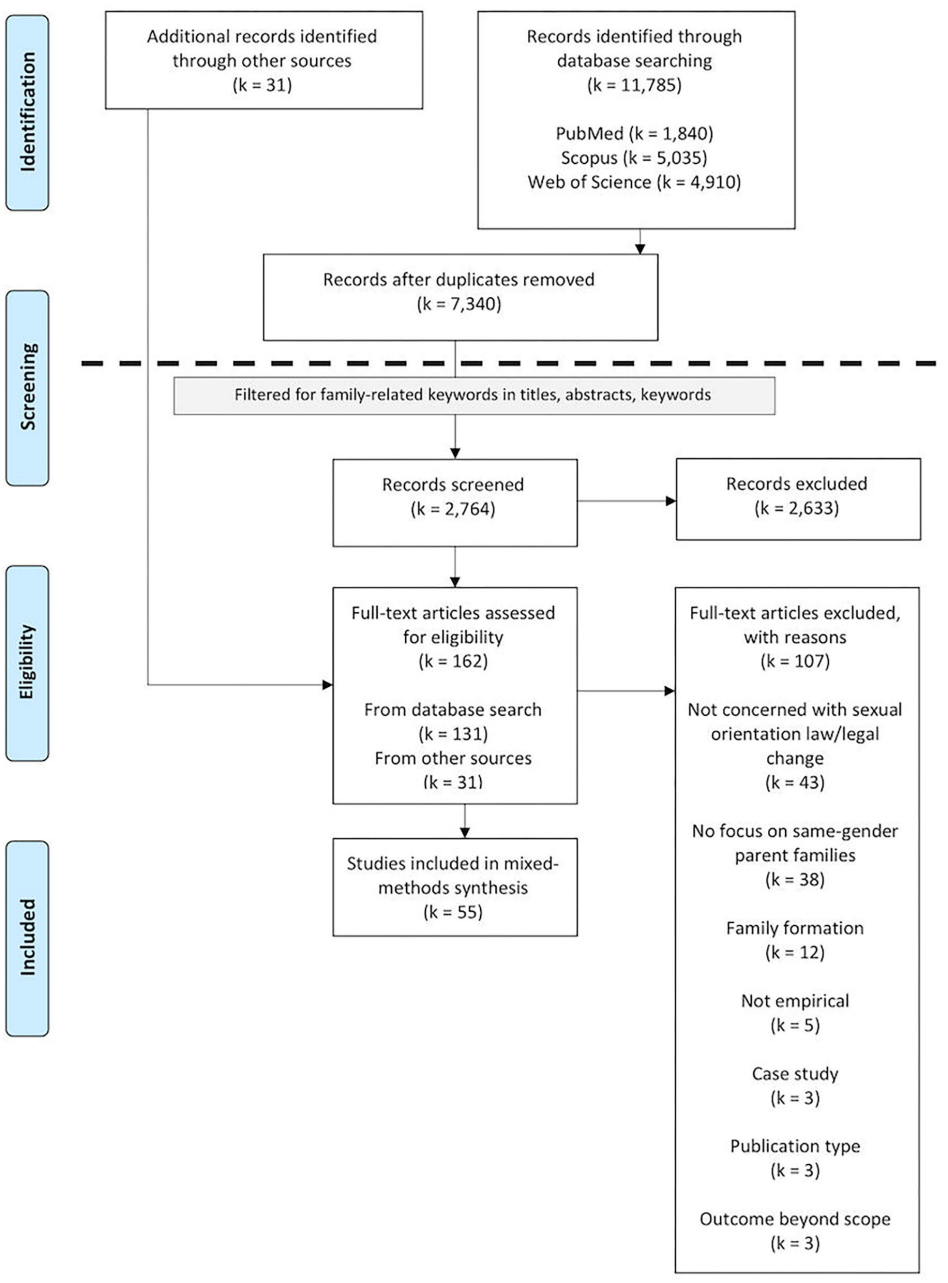
PRISMA flow diagram of the study selection process.

The first and second author piloted the eligibility screening on a random sample of 50 records and then screened another 200 randomly selected records to calculate interrater reliability. Interrater agreement was deemed sufficiently high (94%; κ_Brennan−Prediger_ = 0.88) to ensure a reliable screening of the remaining records (2,514) by one rater (see Figure 1). The record's full text was assessed in case of ambiguous information.

The first two authors independently carried out the fulltext assessments of eligible studies identified via database search (*k* = 131) and subsequently via forward-backward-search (*k* = 31). All discrepancies in final inclusion decisions were resolved via discussion or arbitration by the third author (see OSF-Supplement S2 for references and reasons for exclusion).

### Data Extraction

We developed standardized coding materials (OSF-Supplement S3) to ensure reliable extraction of the following variables: Publication source; methodology; study name and wave (if applicable); year, country, and mode of data collection; sample size and characteristics (e.g., generation [parent, child, both], age, gender, socioeconomic status); family type; type of law; timeframe (current, retrospective, mixed) for qualitative studies; measurement points (cross-sectional, longitudinal, repeated cross-sectional) for quantitative studies; outcome information on measurement and conceptual level.

The first and second author independently coded each study and resolved discrepancies by discussion and verification with original reports. Interrater agreement was excellent (*Md*_%_ = 97.67, range_%_ = 87.27–100; *Md*_κ_ for categorical variables = 0.97, range_κ_ = 0.79–1.00).

#### Extraction of Effect Sizes

We extracted effect sizes on the strength of the association between either legal variation, parental legal status, or country of residence and any of our specified outcomes (i.e., parent and child health, family functioning, child educational attainment) from quantitative studies. Although not preregistered, we also extracted effect sizes on predictors of law-related health outcomes (e.g., predictors of worrying about legal status, Reeves, 2011). As expected, effect sizes were too conceptually dissimilar to be meta-analyzed but are reported for illustrative purposes. Coding information and forest plots are provided in the OSF-Supplements S4 and S5.

### Synthesis of Results and Epistemological Position

#### Thematic Synthesis

We chose a data-based convergent design (Hong et al., 2017) to synthesize primary study results, which has been shown to be particularly suitable for generating frameworks or theories, the overall aim of our review. Here, a single synthesis method is used to synthesize results from both qualitative and quantitative studies by either “qualitizing” quantitative findings (by grouping them into themes) or “quantitizing” qualitative findings (by assigning them numerical values; Sandelowski et al., 2006; Hong et al., 2017).

Specifically, we chose to “qualitize” findings by using thematic synthesis (Thomas and Harden, 2008) based on our preliminary literature search that indicated a large share of studies using a qualitative approach. Our final sample composition (76% qualitative studies) corroborated this choice.

Thematic synthesis follows three steps, namely descriptive line-by-line coding, the generation of descriptive themes, and the generation of analytical themes that go beyond the data (Thomas and Harden, 2008). Based on the substantial number of possibly eligible studies, we employed a purposive sampling approach to derive a pilot sample of studies that served as the basis for developing an initial thematic codebook. To generate a rich set of codes, we aimed to maximize heterogeneity of included studies in this pilot sample with regard to populations, methodology, investigated laws, and outcomes. The pilot sample included the following seven studies: Boertien and Bernardi (2019), Butterfield and Padavic (2014), Goldberg et al. (2013), Hequembourg (2004), Malmquist et al. (2020), Ollen and Goldberg (2016), and Zhabenko (2019).

The first three authors independently performed inductive line-by-line coding of these studies' results sections and generated a first set of codes. During this early stage, we already placed codes within three groups (later defined as our analytical themes): (i) Codes that were concerned with the impact of laws, (ii) codes that were concerned with counteractions families engaged in to mitigate this impact, and (iii) codes that described possible moderators of this impact. For impact-related codes, we specified the valence (positive, negative, no effect). For codes relating to counteractions, we also extracted reasons not to engage in this counteraction or side-effects of this counteraction.

The resulting codebook was circulated among all researchers and iteratively refined. To facilitate integration with existing minority stress and family theories, codes were labeled in line with terminology used within these frameworks where possible.

Consequently, result sections of remaining studies were assigned codes using a deductive-inductive approach. That is, we chose to assign codes based on our initial codebook where possible but allowed for new codes to emerge. In quantitative studies, results from hypothesis tests of interest to our review were assigned codes. In qualitative studies, quotes from individual participants and study authors' interpretations or descriptions of themes were assigned codes. Including individual participants' quotes ensured the inclusion of studies not primarily concerned with the impact of laws or legal recognition or of studies that did not report higher-order themes relevant to the review.

The first author coded all studies and refined the codebook by collapsing or further differentiating codes. Due to the heterogenous sociolegal climate described in these studies, we chose to code every study at the lowest (i.e., the code) level. The refined codebook was again circulated among all researchers.

Separating the impact of sexual orientation laws from the impact of societal prejudice, general legal challenges for stepfamilies, or lacking biological ties to a child posed a challenge during coding. Arguably, the compound impact of these related (but distinct) phenomena makes a differentiation impossible for research participants themselves. We employed a conservative coding strategy and only included quotes and study authors' statements that explicitly referenced the impact of laws or (lacking) legal recognition. While this certainly resulted in a loss of codable data, it strengthens the validity of our results.

The first and second author independently coded 36% of studies according to the final codebook (OSF-Supplement S6). Remaining studies were coded by the first author and the assignment of codes was validated by the second author. Discrepancies were resolved by discussion within the research team.

After completion of coding, we developed and critically discussed broader descriptive themes. While being firmly grounded in primary study results, we assigned theme labels again in line with minority stress and family theories. Lastly, three overarching analytical themes were developed that reflected our initial grouping (i.e., impact-related themes, counteractions, moderators).

#### Negative Case Analysis

We conducted a negative case analysis after completion of coding (Yardley, 2015) to ensure methodological rigor. Here, researchers explicitly search for cases that contradict their theoretical prediction. Empirical evidence and theory (Meyer, 2003; Hatzenbuehler, 2016) predict a positive impact of favorable legal change and, conversely, a negative impact of lacking legal recognition or criminalizing legislation. Thus, we identified coded units concerned with a negative impact of favorable legal change or a positive impact of unfavorable legislation.

#### Epistemological Position

According to the classification of research synthesis by Suri (2013), we adopt a positivist approach, which is characterized by objectivity, rigorous systematization, and empiricism, to conceptualize our systematic review and to derive an overarching model. This is reflected in our preregistered protocol, our exhaustive sampling approach, and our standardized coding materials and procedures. Although our predominantly qualitative evidence base would allow for a more interpretative approach (e.g., Eaves, 2001), we see our model as guiding primarily quantitative, inherently positivist, study designs.

## Results

### Study Characteristics

In all, the present review comprises 55 studies (49 unique samples). Tables 2, 3 report summary statistics of main study characteristics. Individual study characteristics are reported in OSF-Supplements S7 and S8. Included studies were published in peer-reviewed journal articles (*k* = 49), book chapters (*k* = 3), dissertations (*k* = 2), and in a research report (*k* = 1). Forty-two studies used a qualitative design, eight a quantitative, and five a mixed-methods design. Years of data collection (if reported) ranged from 1995 to 2018 (*Md* = 2012).

**Table 2:** Study characteristics related to sociodemographics; overall and stratified by investigated generation.

**Variable**	**Overall (*k* = 55)**	**Parent (*k* = 47)**	**Child (*k* = 6)**	**Both generations (*k* = 2)**
**Family type**				
Mixed (planned and stepfamilies)	28	22	5	1
Planned	17	16	1	–
Stepfamilies	1	1	–	–
Not reported/unclear	9	8	–	1
***N***^**a**^				
Overall (*M;* Range)	1,958,088 (39,961; 6–1,952,490)	4,470 (106; 6–732)	1,952,839 (390,568; 8–1,952,490)	779 (390; 37–742)
Excl. controls (*M;* range)	13,195 (269; 6–7,792)	4,275 (102, 6–732)	8,141 (1,628; 8–7,792)	779 (390; 37 – 742)
***N***^**a**^ **(without Boertien and Bernardi**, **2019****)**				
Overall (*M*, range)	5,598 (117, 6–742)	4,470 (106, 6–732)	349 (87, 8–153)	779 (390, 37–742)
Excl. controls (*M;* range)	5,403 (113; 6–742)	4,275 (102, 6–732)	349 (87, 8–153)	779 (390, 37–742)
**Gender**				
Female	24	24	–	–
Male	1	1	–	–
Mixed	30	22	6	2
**Parent education**				
Predominantly^b^ well-educated	22	21	–	1
Not predominantly well-educated	3	3	–	
Unclear/not reported	24	23	–	1
**Parent ethnicity**				
Predominantly^b^ white/European American/Caucasian	29	27	–	2
Not predominantly White/European American/Caucasian	4	4	–	
Unclear/not reported	16	16	–	–
**Child age group**				
Children (0–18)	2	–	2	–
Adults (18+ years)	2	–	1	1
Mixed	4	–	3	1

*Cell entries indicate number of studies (excepting rows reporting sample sizes)*.

a *Based on unique samples (k = 49). In case of studies reporting subsamples of other included studies (e.g., Goldberg and Allen, 2013, reports on a subsample of Goldberg and Kuvalanka, 2012) full sample size was used in sample size calculations. In case of studies reporting on participants with and without children (e.g., Rostosky et al., 2016), sample size for participants with children (i.e., parents) was used for sample size calculation*.

b *> 75% or described as such by primary study authors (for education: 75% of sample at least some college education). Parent education and ethnicity not coded for studies investigating the child generation*.

**Table 3:** Study characteristics regarding investigated laws and timeframe.

**Variable**	**Overall (*k* = 55)**
**Law type**	
General legal situation for same-sex parent families	24
Adoption (general and second-parent)	12
Marriage and civil union	9
Country of data collection (proxy)	3
Marriage and civil union; anti-discrimination laws^a^	1
Adoption (general and second-parent); Anti-discrimination laws	1
Composite score of legal climate	1
Criminalization	1
Anti-discrimination laws	1
Other^b^	2
**Timeframe (qualitative studies/qualitative part of mixed methods study)**	
Current	21
Retrospective	11
Mixed	14
**Study design (quantitative studies/quantitative part of mixed methods study)**	
Cross-sectional	9
Longitudinal	1
Repeated cross-sectional	1

a *Sensitivity analysis*.

b *Same-sex marriage ban (Rostosky et al., 2010); legal status per parent (Reeves, 2011). k = 46 for qualitative timeframe because qualitative part of Kosciw and Diaz (2008) not coded. k = 11 for quantitative study design because quantitative part from Stambolis-Ruhstorfer and Descoutures (2020) and Chamberlain et al. (2015) not coded*.

Almost two thirds of studies were conducted in the US (*k* = 35), 11 in one or more European countries (Belgium, Bulgaria, Croatia, Czech Republic, France, Hungary, Italy, Poland, Slovakia, Slovenia, Spain, Sweden, UK), four in Australia and New Zealand, and one in Canada and Russia each. Three studies reported cross-country comparisons between the US and the Netherlands (Bos et al., 2008), the US and Canada (Shapiro et al., 2009), and Canada and France (Vyncke and Julien, 2007). In line with our protocol, we included these studies because of their explicit reference to the (socio-)legal climate as a possible cause of the investigated group differences. However, we caution against interpreting findings from these studies as direct evidence of the impact of legislation, as they lack an explicit operationalization.

### Thematic Synthesis

#### Descriptive Code Statistics

Our final database comprised 633 coded units of data (*M* = 11.51, *SD* = 9.67, range = 1–50 per study). Taking into account multiple assignments of codes in one study, this amounted to 458 individual codes (*M* = 8.33, *SD* = 5.7, range = 1–22 per study). Thirty-five coded units stemmed from quantitative studies, from which we extracted 68 effect sizes (see OSF-Supplements S4 and S5 for numerical results and forest plots).

We identified 50 codes that we grouped into 13 descriptive themes and three analytical themes (see Table 4 for code frequencies and OSF-Supplement S9 for a correlation matrix). Detailed code descriptions and citation examples can be found in the OSF-Supplement S10. The most frequent codes (code and theme labels italicized) across studies were *Legal-Financial Security* (coded in 47% of studies), *(Frustration with) Discrimination and Unequal Treatment* (45%), and *Legal Documents and Paper Trails* and *Reaction of Others* (40% each). Six codes were only coded in one study each (2%): *Acquiescence, Creation of Emotional Dependency, Creation of Financial Dependency, Emotion Regulation (Self)* (all Butterfield and Padavic, 2014), *Relationship with Wider Social Network* (Vyncke and Julien, 2007), and *School Progress* (Boertien and Bernardi, 2019).

**Table 4:** Frequencies of assigned codes across studies as well as corresponding descriptive and analytical themes and model pathways.

**Number**	**Code**	**Descriptive theme**	**Analytical theme**	**n (%)**	**%_**no effect**_**	**Model pathway**
I-E-1	School progress	Education	Impact	1 (1.82)	100	F.9
I-F-1	Division of labor (parenting tasks)	Family	Impact	9 (16.36)	18.18	F.3
I-F-2	Family legitimacy and cohesion	Family	Impact	21 (38.18)	6.45	F.1
I-F-3	Interparental relationship	Family	Impact	9 (16.36)	33.33	P.6/F.2
I-F-4	Parent-child relationship	Family	Impact	14 (25.45)	26.09	F.7
I-F-5	Parental legitimacy	Family	Impact	17 (30.91)	12	F.6
I-F-6	Relationship with family of origin	Family	Impact	7 (12.73)	22.22	P.2
I-F-7	Relationship with wider social network	Family	Impact	1 (1.82)	100	P.2
I-F-8	Sibling relationship	Family	Impact	2 (3.64)	33.33	F.8
I-G-1	False panacea	General	Impact	13 (23.64)		P.1/P.5
I-G-2	No impact	General	Impact	2 (3.64)	100	–
I-H-1	Health and well-being	Health, well-being, and security	Impact	14 (25.45)	24	P.2/F.9
I-H-2	Hypervigilance	Health, well-being, and security	Impact	7 (12.73)	0	P.2
I-H-3	Legal-financial security	Health, well-being, and security	Impact	26 (47.27)	0	P.2
I-H-4	Perceived powerlessness	Health, well-being, and security	Impact	5 (9.09)	0	P.2
I-M-1	(Frustration with) discrimination and unequal treatment	Minority stress	Impact	25 (45.45)	10.64	P.1/P.5
I-M-2	(Legal) rejection sensitivity	Minority stress	Impact	15 (27.27)	0	P.1/P.5
I-M-3	Sexual orientation concealment	Minority stress	Impact	10 (18.18)	16.67	P.1/P.5
I-S-1	Backlash	Safety concerns	Impact	2 (3.64)	0	P.2
I-S-2	Constant concerns	Safety concerns	Impact	10 (18.18)	0	P.2
I-S-3	Family cohesion	Safety concerns	Impact	18 (32.73)	3.03	P.2
I-S-4	Health and well-being	Safety concerns	Impact	16 (29.09)	11.11	P.2
I-S-5	Legal-financial	Safety concerns	Impact	11 (20)	0	P.2
I-S-6	Physical symptoms	Safety concerns	Impact	2 (3.64)	0	P.2
C-F-1	Acquiescence	Within family	Counteraction	1 (1.82)		C.2
C-F-2	Creation of emotional dependency	Within family	Counteraction	1 (1.82)		C.2
C-F-3	Creation of financial dependency	Within family	Counteraction	1 (1.82)		C.2
C-F-4	Creation of legal dependency	Within family	Counteraction	17 (30.91)		C.2
C-F-5	Emotion regulation (others)	Within family	Counteraction	4 (7.27)		C.2
C-F-6	Parenting practices	Within family	Counteraction	2 (3.64)		C.2
C-P-1	Emotion regulation (self)	Within person	Counteraction	1 (1.82)		C.1
C-P-2	Information seeking	Within person	Counteraction	5 (9.09)		C.1
C-P-3	Overcoming heteronormativity	Within person	Counteraction	5 (9.09)		C.1
C-S-1	Activation of community accountability	Within system	Counteraction	7 (12.73)		C.3
C-S-2	Activism	Within system	Counteraction	8 (14.55)		C.3
C-S-3	Concealment	Within system	Counteraction	5 (9.09)		C.3
C-S-4	Legal documents and paper trails	Within system	Counteraction	22 (40)		C.3
C-S-5	Opposing/ignoring legal limitations	Within system	Counteraction	5 (9.09)		C.3
C-S-6	Relocation	Within system	Counteraction	8 (14.55)		C.3
C-S-7	Symbolism	Within system	Counteraction	17 (30.91)		C.3
M-CF-1	Anecdotal evidence and role models	Contextual factors	Moderator	7 (12.73)		M.1
M-CF-2	Reaction of others	Contextual factors	Moderator	22 (40)		M.1
M-CF-3	Saliency of legal recognition	Contextual factors	Moderator	18 (32.73)		M.1
M-CC-1	Couple gender	Couple characteristics	Moderator	4 (7.27)		M.3
M-CC-2	Socioeconomic status	Couple characteristics	Moderator	11 (20)		M.3
M-F-1	Family member	Family characteristics	Moderator	9 (16.36)		M.2
M-F-2	Family type	Family characteristics	Moderator	6 (10.91)		M.2
M-I-1	Child characteristics	Individual differences	Moderator	5 (9.09)		M.4
M-I-2	Legal awareness	Individual differences	Moderator	2 (3.64)		M.4
M-I-3	Parental characteristics	Individual differences	Moderator	8 (14.55)		M.4

*n = number of studies the code occurred in, % = proportion of studies (out of 55) a code occurred in. %_noeffect_ = proportion of coded statements that indicated no effect out of all statements pertaining to this code (impact codes only; no direction coded for False Panacea). Note that codes may have been assigned more than one time in the respective primary studies*.

#### Descriptive and Analytical Themes

See OSF-Supplement S10 for detailed descriptions of codes and themes. The essence of our thematic synthesis is summarized below.

The analytical theme *Impact* (376 coded units) incorporates themes related to the impact of sexual orientation laws, most often those that regulate the legal recognition of family relationships. These themes relate to the impact on aspects of family life and relationships (e.g., division of parenting tasks, feelings of parental and family legitimacy, family relationships; descriptive theme *Family*), predictors and outcomes related to the health and well-being of family members (*Health, Well-Being, and Security; Minority Stress; Safety Concerns*), and child educational outcomes (*Education*). This analytical theme also incorporates the perception of no impact of legislation on any area of life or the insufficient or even detrimental consequences of (positive) legal change (*General*). It should be noted that only in two qualitative studies (Ollen and Goldberg, 2016; Malmquist et al., 2020), participants expressed that lacking legal recognition did not impact any area of their or their family members' life (see also Table 4). In both cases, the coded units from these studies referred to children.

The analytical theme *Moderator* (122 coded units) describes how some families or family members are impacted more than others due to contextual (*Contextual Factors*), familial (*Family Characteristics*), couple-level (*Couple Characteristics*), and individual factors (*Individual Differences*).

The analytical theme *Counteraction* (135 coded units) is concerned with the numerous counteractions on the personal (*Within Person*), familial (*Within Family*), and systemic (*Within System*) level that same-sex parent families engage in to alleviate the impact of sexual orientation laws on their relationships, their financial and legal security, and their health and well-being.

#### Negative Case Analysis

We identified eleven coded units from nine studies (3% of impact statements) in our negative case analysis (see OSF-Supplement S11). These include losing financial benefits due to legal change (e.g., single parent benefits, *n* = 2), growth of resilience, pride, and an improved parent-child relationship due to legal disadvantages (*n* = 2), negative effects of increased outness due to marriage or a positive effect of total concealment (*n* = 3), marginalization of same-sex couples not wanting to marry (*n* = 2), and exacerbated custody disputes due to a formal relationship recognition (*n* = 2).

### The Legal Vulnerability Model for Same-Sex Parent Families

Our thematic synthesis identified (i) pathways through which sexual orientation laws might impact key predictors and outcomes of parental and child health and family functioning, (ii) factors that may moderate this association, and (iii) counteractions that family members engage in to mitigate these effects. We now integrate these findings with existing empirical work and theories from the field of minority stress and family research (see below) and propose a conceptual, empirically testable model of legal vulnerability for same-sex parent families. Given the mostly qualitative or mixed methods evidence (85%) and the heterogeneous or insufficient operationalizations of legal status or sexual orientation laws in quantitative studies (e.g., using country of data collection as a proxy), we emphasize the hypothesis-generating nature of our model.

**Figure 2:**
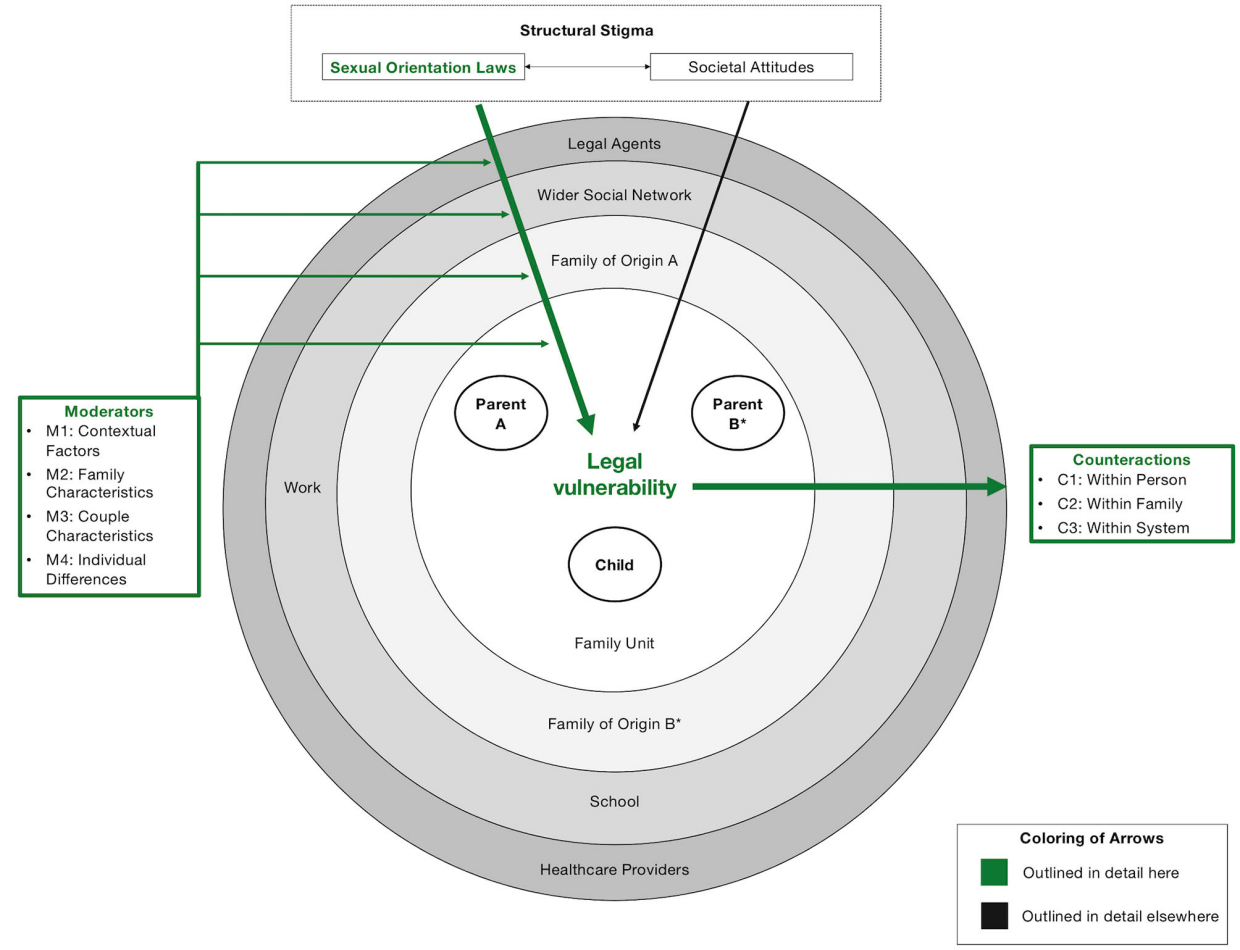
The legal vulnerability model for same-sex parent families. Colored arrows (green) are outlined in detail here and depict empirically testable relations between constructs, not evidence strength. ^*^Nonlegal status to (grand-)child and/or partner.

**Figure 3:**
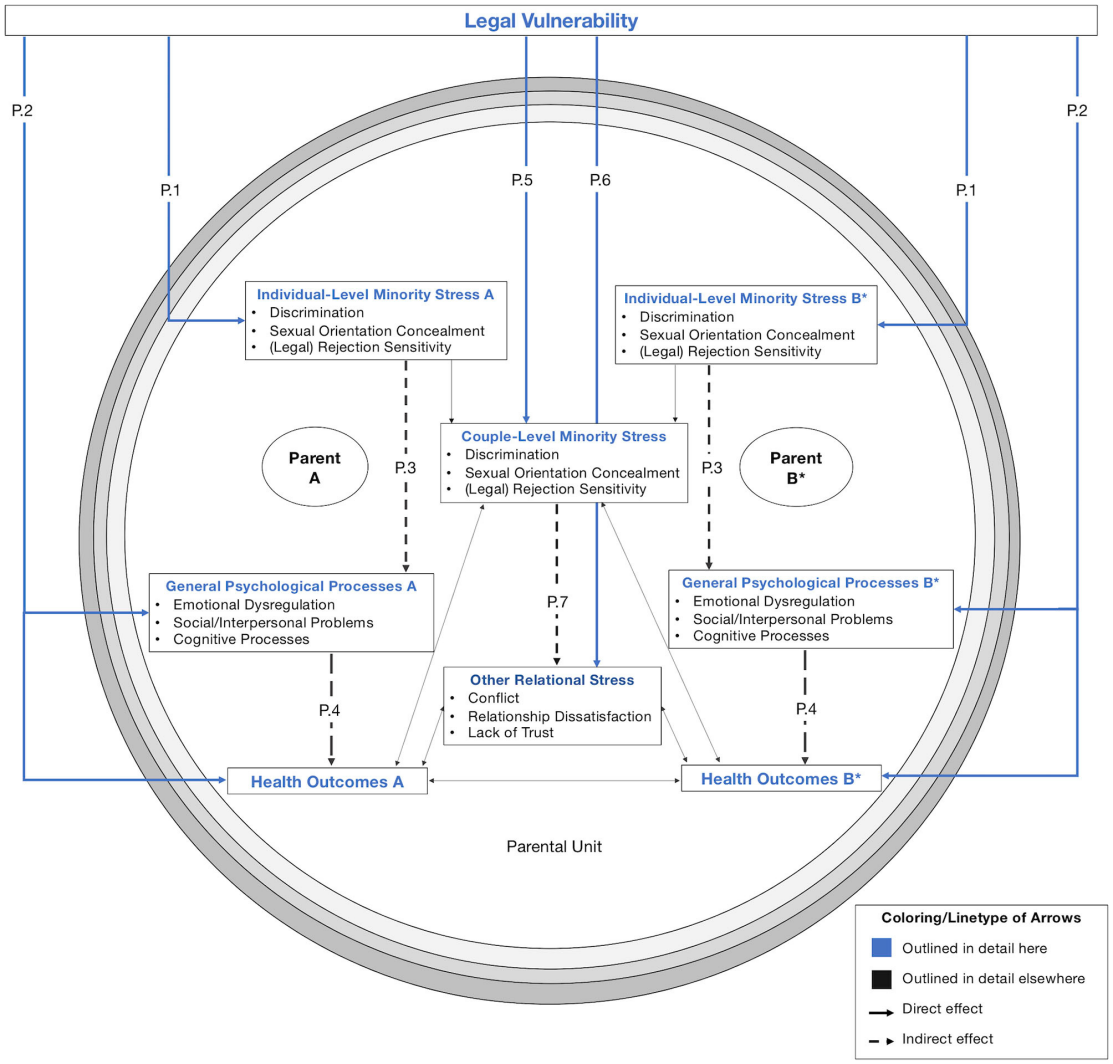
Impact of legal vulnerability on parental health. Colored arrows (blue) are outlined in detail here and depict empirically testable relations between constructs (highlighted with colored headings), not evidence strength. Dashed lines depict indirect effects of legal vulnerability (i.e., through other constructs). Thin, unlabeled arrows (e.g., between other relational stress and health outcomes) depict interrelations between constructs outlined in detail elsewhere. Note that legal vulnerability is depicted outside the parental unit for clarity. Terminology was chosen in line with Feinstein (2020), Hatzenbuehler (2009), LeBlanc et al. (2015), and Meyer (2003). ^*^Nonlegal status to child and/or partner.

**Figure 4:**
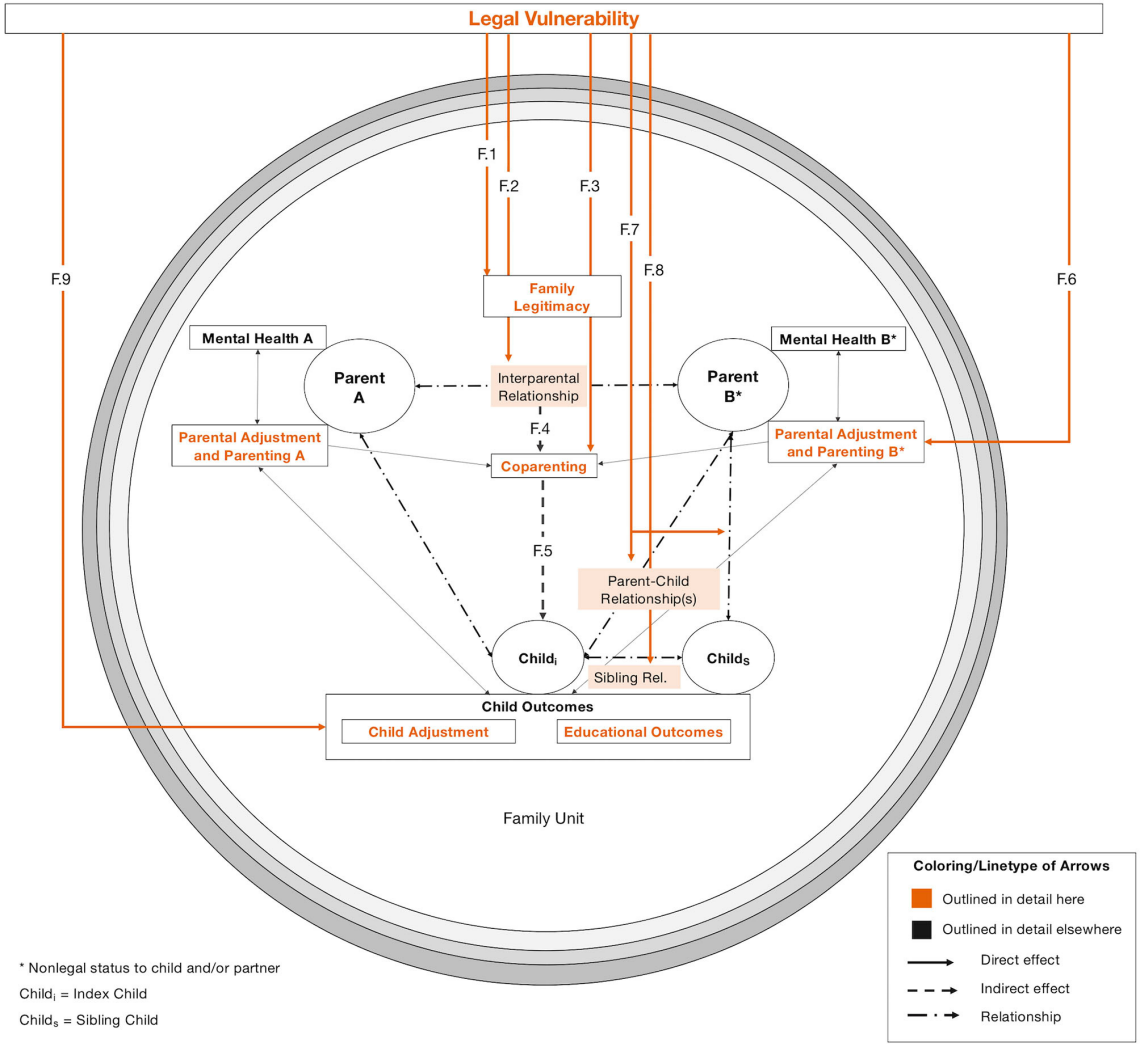
Impact of legal vulnerability on family functioning and child outcomes. Colored arrows (orange) are outlined in detail here and depict empirically testable relations between constructs (highlighted with colored headings), not evidence strength. Dashed lines depict indirect effects of legal vulnerability (i.e., through other constructs). Thin, unlabeled arrows (e.g., between parental adjustment and coparenting) depict interrelations between constructs outlined elsewhere. Boxes colored in orange as well as dot-dashed-lines depict relationships between family members. Note that legal vulnerability is depicted outside the family unit for clarity. Terminology was chosen in line with Feinberg (2003).

**Figure 5:**
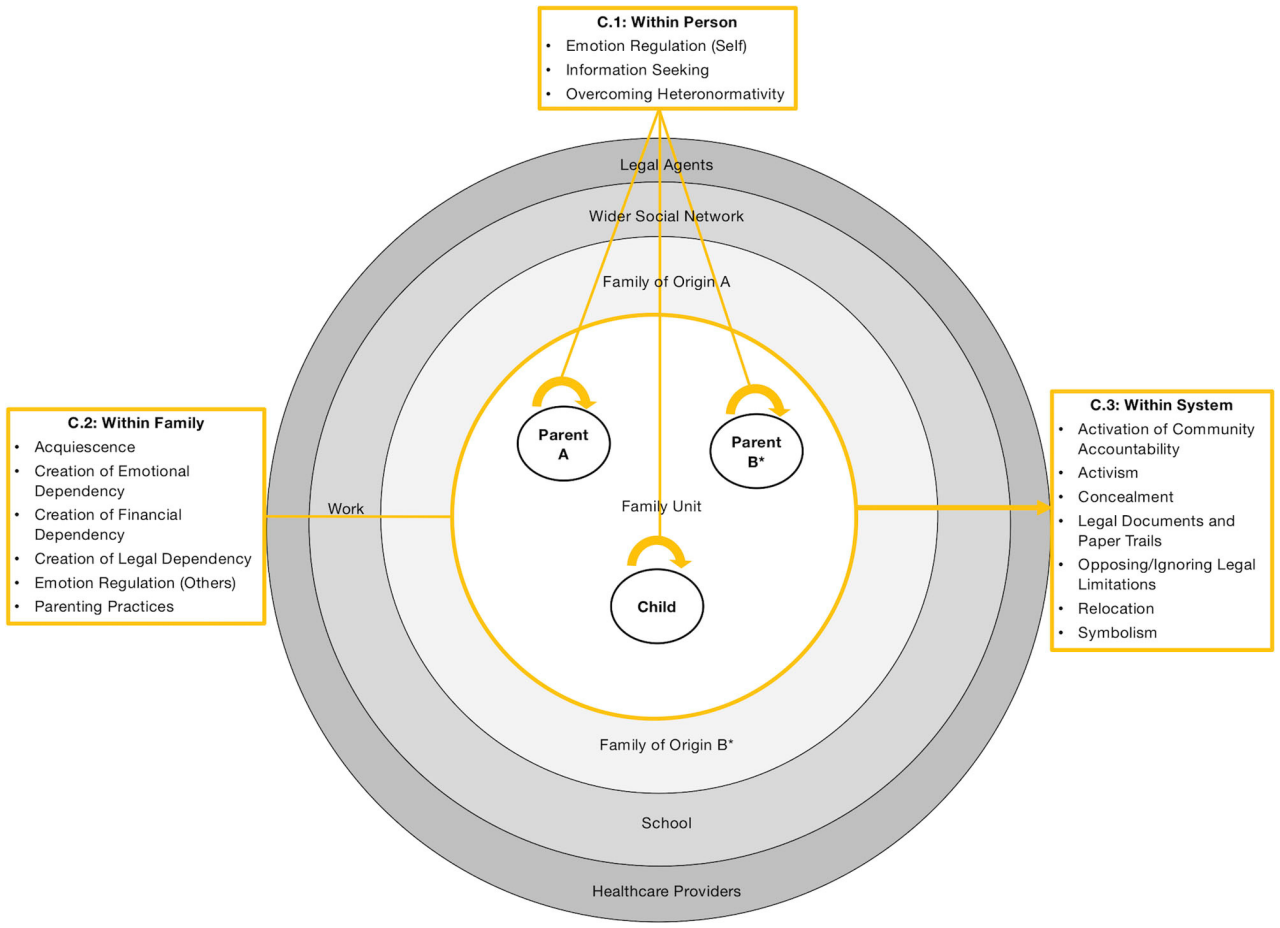
Counteractions to alleviate the impact of legal vulnerability on the personal, familial, and systemic level. ^*^Nonlegal status to child and/or partner.

Our model is illustrated in Figures 2–5: Figure 2 depicts our overall model from a socio-ecological perspective (Bronfenbrenner, 1986). Here, we propose that sexual orientation laws^2^ create actual and perceived *legal vulnerability* (a novel concept; see section “Legal Vulnerability”) in all members of the family unit (innermost circle), which impacts parental and child health and family relationships in reciprocal ways (detailed in Figures 3, 4). In Figure 5, we illustrate the counteractions that same-sex parent families engage in to mitigate the impact of legal vulnerability on themselves or family members.

### Theoretical Foundations of the Legal Vulnerability Model

The legal vulnerability model is based on minority stress and family theories, which we briefly summarize below.

#### Minority Stress Theory

Minority Stress Theory (Meyer, 2003) posits that sexual minorities face unique, chronic, and socially based stressors due to their societally marginalized status. These stressors account for their heightened vulnerability to experiencing adverse (mental) health outcomes (e.g., Lick et al., 2013; Plöderl and Tremblay, 2015). Located on a distal-proximal continuum, minority stressors include stressful events such as experiencing discrimination, but also behaviors and cognitions such as internalized homonegativity, expectations of rejection, or sexual orientation concealment (Meyer, 2003). Importantly, minority stress theory and its application in therapeutic practice (Pachankis, 2015) assume minority stress to affect shared pathways across disorders which are susceptible to stress.

#### The Psychological Mediation Framework

The Psychological Mediation Framework (Hatzenbuehler, 2009) elucidates psychological pathways through which minority stressors impact (mental) health outcomes. It posits that general psychological processes, such as emotional dysregulation (e.g., rumination, hypervigilance), social problems (e.g., isolation), and maladaptive cognitive processes and schemas (e.g., hopelessness) mediate the association between minority stressors and (mental) health.

#### The Rejection Sensitivity Model

The Rejection Sensitivity Model (Feinstein, 2020) complements minority stress theory and the psychological mediation framework by formalizing rejection sensitivity as a (proximal) minority stressor. It is theorized to affect mental health via a combination of cognitive (i.e., expecting rejection, interpreting ambiguous situations as evidence of rejection) and affective processes (i.e., anxiety or anger about experiencing future rejection).

#### The Couple-Level Minority Stress Model

The Couple-Level Minority Stress Model (LeBlanc et al., 2015) extends minority stress theory by stipulating that individual minority stressors have an equivalent on the couple level. These include discrimination due to being visible as a couple (i.e., experiences of discrimination), beliefs about the relationship being less valuable (i.e., internalized homonegativity), or concealing the romantic nature of the relationship in public (i.e., sexual orientation concealment; LeBlanc et al., 2015). The model also considers dyadic minority stress processes between partners, including minority stress discrepancies, contagion, and proliferation (LeBlanc et al., 2015).

#### Family Systems Theory

Family Systems Theory conceptualizes the family as an organized whole, where family members (and consequently their behaviors, cognitions, and emotions) are mutually interdependent (Minuchin, 1974). Thus, from a systemic perspective, the behaviors and well-being of an individual family member can only be understood in relation to their location within the family system and its interrelations.

#### The Coparenting Model

The Coparenting Model (Feinberg, 2003) provides a formalization of coparenting (i.e., the cooperation, coordination, and mutual support in child rearing by parents) as a central executive subsystem of the family. It comprises four coparenting dimensions, namely joint family management, division of labor, childrearing agreement, and supporting/undermining the partner, as well as individual (e.g., parental self-efficacy), familial (e.g., the interparental relationship), and ecological predictors (e.g., financial resources) of coparenting.

### Legal Vulnerability

Several studies in our review (e.g., Goldberg and Kuvalanka, 2012; Butterfield and Padavic, 2014; Acosta, 2017; Gash and Raiskin, 2018) used the term “legal vulnerability” to describe the precarious legal situation for same-sex parent families. However, to our knowledge, a formal definition of this concept is currently lacking.

Therefore, we offer a working definition of legal vulnerability for same-sex parent families^3^: Legal vulnerability is a heightened and stable risk for family members of expecting or experiencing adverse general and minority-specific outcomes related to health and family functioning due to the (i) lacking legal recognition of family relationships, (ii) lacking protection against discrimination, or (iii) criminalization of the parents' sexual orientation.

This working definition emphasizes four important characteristics of legal vulnerability: First, its influence on the family unit is defined as enduring and stable, rather than as instantaneous. Second, in line with family system theory (Minuchin, 1974), it emphasizes its interrelated (yet varying) effects on all members of the family, including children, and on the family system as an organized whole. Third, it is conceptualized as impacting both general and minority-specific outcomes, which results in a compound impact of legal vulnerability. Fourth, it explicitly includes the anticipation of risk or threat, thereby incorporating the role of (maladaptive) future-oriented cognitive patterns (Roepke and Seligman, 2016) and ruminative tendencies (Nolen-Hoeksema et al., 2008) for mental health. Importantly, we adopt an equifinal approach and do not systematically stratify by types of laws unless explicitly discussed.

### Impact of Legal Vulnerability on Parental Health

Figure 3 depicts the impact of legal vulnerability on parental health by linking general and minority-specific processes in the creation of adverse parental health outcomes that operate through pathways on the individual (P.1, P.2, P.3, P.4) and the couple level (P.5, P.6, P.7). Individual minority stressors and general psychological processes for each parent are depicted on both sides of the model (i.e., mirroring each other). Shared processes between parents (i.e., couple-level minority stress and other relational stress) are depicted in the figure center. Health outcomes for both parents are depicted as primary outcomes in the bottom part.

Our empirical evidence base and the theoretical foundations of our model would allow for a focus on mental health. We chose to use the general term “health” for two reasons: First, physical health disparities and physical health correlates of minority stress are commonly conceptualized as sequelae of heightened (minority) stress exposure (Lick et al., 2013; Flentje et al., 2020). Second, lacking access to legally recognized parental relationships can lead to material and financial disadvantages for same-sex couples (e.g., lack of partner insurance or fiscal benefits), which are well-established health-related risk factors (Pampel et al., 2010; Phelan et al., 2010).

#### Pathways on the Individual Level

We propose that legal vulnerability exacerbates well-established minority stressors (e.g., discrimination, sexual orientation concealment, and rejection sensitivity; P.1) but also constitutes an independent minority stressor that impacts health-related outcomes and mediating psychological processes directly (P.2). Pathways P.3 and P.4 depict these mediational pathways (as theorized in the Psychological Mediation Framework; Hatzenbuehler, 2009) that link minority stress and (mental) health. These mechanisms are discussed in detail elsewhere (Hatzenbuehler, 2009) but included for the sake of completeness. Similarly, associations between sexual orientation laws and minority stress or mental health outcomes on the individual level (i.e., unrelated to parental status) are not the focus of this model but are hypothesized to impact parental health (as described elsewhere, e.g., Hatzenbuehler et al., 2010; Berg et al., 2013; Pachankis and Bränström, 2018).

*P.1: Minority Stress*. Legal vulnerability adds structural facets to well-established minority stressors such as discrimination, sexual orientation concealment, and rejection sensitivity. With regard to discrimination, evidence within our review suggests that members of same-sex parent families experience various discriminatory instances within the legal system or as a consequence of lacking legal recognition of family relationships (e.g., Kazyak, 2015; Park et al., 2016; Gash and Raiskin, 2018), as well as feelings of unequal treatment (e.g., Goldberg et al., 2013; Maxwell and Kelsey, 2014; Bacchus, 2018).

We found evidence that legal vulnerability can lead to concealment of the parents' sexual orientation or the parental role (e.g., Sobočan, 2013; Messina and D'Amore, 2018; Zhabenko, 2019), particularly so in hostile or criminalizing environments. In these instances (see our negative case analysis) concealment was sometimes preferred over the risks to personal safety associated with living authentically (Denman, 2016; Zhabenko, 2019). This adds to our understanding of the multifaceted nature of sexual orientation concealment, which can be beneficial to sexual minorities in highly stigmatizing environments (Pachankis et al., 2020). Some evidence in our review also suggests that legal vulnerability is associated with an increased selectivity in sexual orientation concealment, with same-sex parent families remaining open to their families of origin, but less so to people in the wider social network (Vyncke and Julien, 2007; Vučković Juroš, 2019b; Zhabenko, 2019). Conversely, decreased legal vulnerability (e.g., through a recognized parental relationship) may lead to increased outness and visibility as a member of a same-sex parent family (e.g., through being visible as a married couple).

Legal vulnerability also adds a structural facet to rejection sensitivity (Feinstein, 2020). Based on the evidence provided in our review, we propose that legal vulnerability is associated with rejection sensitivity toward the legal system (i.e., legal rejection sensitivity): Within our evidence base, this legal rejection sensitivity took the form of (anxiously) expecting that legal documents will not hold up in court (e.g., McClellan, 2001; Bergen et al., 2006; Goldberg et al., 2013), expectations of prejudicial treatment by actors within the legal system or the state (e.g., Goldberg et al., 2014; Gash and Raiskin, 2018; Wheeler et al., 2018; Zhabenko, 2019), distrust in the state or foreign jurisdictions recognizing the family structure (e.g., when traveling; Bergen et al., 2006; Gartrell et al., 2019), or questioning the motivation behind (e.g., Rawsthorne, 2013) or the permanency of progressive legal change (i.e., expecting a backlash; Goldberg et al., 2013; Denman, 2016).

Similar to individual rejection sensitivity (Feinstein, 2020), we propose that legal rejection sensitivity incorporates cognitive and affective components. The instances (i.e., the rejection within the legal system or its actors) outlined above are not only expected, but also anxiously anticipated. Pervasive safety concerns found within our review illustrate this anxious expectation of legal rejection that members in same-sex parent families experience due to their legal vulnerability (e.g., Shapiro et al., 2009; Rostosky et al., 2010; Goldberg et al., 2013; Kazyak, 2015; DiGregorio, 2016; Zhabenko, 2019). Conversely, strong institutional support through anti-discrimination laws and legal recognition of relationships might reduce legal rejection sensitivity, as evidence from our review suggests (Vučković Juroš, 2019b).

We did not find evidence for a link between legal vulnerability and internalized homonegativity (see Reeves, 2011, for the only and non-significant association). However, internalized homonegativity bears resemblance to feelings of decreased parental legitimacy due to lacking legal validation (e.g., Butterfield and Padavic, 2014; Malmquist, 2015; Gash and Raiskin, 2018). Specifically, negative societal messages about parenting capabilities (and rights) of same-sex parents could be internalized, which may lead to similar feelings of guilt and shame or other adverse mental health outcomes as internalized homonegativity on the individual level (Newcomb and Mustanski, 2010).

*P.2: General Psychological Processes and Health Outcomes*. We found evidence that legal vulnerability targets psychological processes (e.g., rumination or hypervigilance, social problems, maladaptive cognitive processes such as hopelessness) theorized to mediate the association between minority stress and (mental) health (Hatzenbuehler, 2009).

Ruminative tendencies are reflected in various and consuming safety concerns that parents experience as a consequence of their legal vulnerability. Within our review, these included concerns about the family's cohesion (e.g., Hequembourg and Farrell, 1999; McClellan, 2001; Bergen et al., 2006; Rawsthorne, 2013; Gash and Raiskin, 2018; Zhabenko, 2019), legal-financial security (e.g., Shapiro et al., 2009; Rostosky et al., 2010; Reeves, 2011), and their own or their family members' well-being (e.g., Padavic and Butterfield, 2011; Malmquist, 2015; Zhabenko, 2019).

Legal vulnerability is also associated with hypervigilance because of the non-recognized, ambiguous, or criminalized legal status of the family (members). This hypervigilance manifested itself in diverse ways within our review, for example by always having important legal documents at hand (e.g., Park et al., 2016; Gash and Raiskin, 2018; Gartrell et al., 2019; Zhabenko, 2019), being vigilant about when and where to publicly display the family structure (Vučković Juroš, 2019b; Zhabenko, 2019), or about a possible relationship dissolution that could entail loss of contact to a non-legal child (Butterfield and Padavic, 2014).

Legal vulnerability might also be associated with social support from people outside the family unit, particularly the family of origin, as evidence suggests (e.g., Hequembourg and Farrell, 1999; Hequembourg, 2004; Zamperini et al., 2016). This association was also found in other qualitative studies on the impact of marriage legislation, interestingly in all directions (i.e., increased support, continued support, continued non-acceptance; Rothblum et al., 2011; Kennedy et al., 2018; Riggle et al., 2018).

Lastly, legal vulnerability might amplify maladaptive cognitive processes such as feelings of invisibility, hope- and powerlessness. In studies within our review, these feelings were directed toward the state or legal system (e.g., Gash and Raiskin, 2018), but also toward the family unit, for example in non-legal parents with regard to parenting decisions (Butterfield and Padavic, 2014), or after a relationship dissolution (that results in a loss of contact with the legally unrecognized child, Kazyak, 2015).

The evidence found in our review also points to direct effects on parental health (but see Vyncke and Julien, 2007). Apart from the various safety concerns same-sex parent families experience, this includes feelings of stress, anger, frustration, and fear due to legally not recognized relationships or dealing with a discriminatory legal system (e.g., Ross et al., 2005; Butterfield and Padavic, 2014; Goldberg et al., 2014; Chamberlain et al., 2015; Kazyak, 2015). Conversely, we found that a positive legal shift is associated with positive emotions (e.g., relief, increased feelings of security; Short, 2007; Goldberg et al., 2013, 2014).

The economic consequences of sexual orientation legislation on the individual (Ash and Badgett, 2006) or the macro-level (Badgett et al., 2019) are beyond the scope of our review. However, we propose that legal vulnerability impacts parental health indirectly through material and financial burdens. The impact of legal vulnerability on the family's legal-financial security was indeed the most frequently assigned code in our review (found in 47% of studies). The health-related benefits of economic well-being are well-documented (Pampel et al., 2010; Phelan et al., 2010), and we propose that access to a partner's insurance, property, or inheritance via legally recognized relationships might be similarly beneficial for parental health.

#### Pathways on the Couple Level

We propose that legal vulnerability also impacts parental health through minority-specific (P.5) and general (P.6) psychological processes that parents experience jointly, as well as through mediating pathways linking minority stress to health outcomes (P.7). To conceptualize these effects, we draw on the couple minority stress model (LeBlanc et al., 2015).

*P.5: Couple Level Minority Stress*. We propose that individual minority stressors as a consequence of legal vulnerability also have an equivalent on the couple level. Specifically, these correspond to the minority stressors outlined in pathway P.1. We argue that they target parents both as individuals and as joint members of the parental unit, as the experience of them is contingent on their parental role.

*P.6: General Relational Stress*. In our review, we found preliminary evidence that legal vulnerability impacts general relational stress, for example due to conflicts that result out of (legal) power differentials between parents (Butterfield and Padavic, 2014). We discuss these mechanisms in section Impact of Legal Vulnerability on Family Functioning and Child Outcomes, where family relations are conceptualized as outcomes in their own right.

Of note, no study in our review directly addressed the impact of legal vulnerability on the mediating role of minority stress in impaired relationship functioning or general relational stress (P.7). We include this pathway for the sake of completeness (see LeBlanc et al., 2015; Cao et al., 2017) and to guide future research questions.

### Impact of Legal Vulnerability on Family Functioning and Child Outcomes

Figure 4 illustrates how legal vulnerability impacts several interrelated areas of family functioning, including subjective and perceived family legitimacy (F.1), family relationships (F.2, F.7, F.8), coparenting (F.3), and parental (F.6) and child adjustment (F.9). Constructs pertaining to individual family members (i.e., parental mental health, parental adjustment and parenting, child adjustment and educational outcomes) are depicted in boxes next to respective family members. Constructs pertaining to all family members (i.e., family legitimacy, coparenting) are depicted in the figure center. Relationships are depicted as dot-dashed lines.

#### F.1: Family Legitimacy

Our review suggests that the legal recognition of the interparental or the parent-child relationship is associated with increased feelings of family legitimacy, stability, normalcy, and cohesion for all family members (Short, 2007; Porche and Purvin, 2008; Goldberg and Kuvalanka, 2012; DiGregorio, 2016; Gash and Raiskin, 2018; Malmquist et al., 2020), and also in the perception of others (e.g., Hequembourg and Farrell, 1999; Rawsthorne, 2013; Vučković Juroš, 2019b; Stambolis-Ruhstorfer and Descoutures, 2020). Conversely, lacking legal recognition has been described as being associated with feelings of diminished family legitimacy, notably only with regard to others, in studies within our review (Goldberg and Allen, 2013; Gash and Raiskin, 2018).

#### F.2: Interparental Relationship

We propose that legal vulnerability impacts the interparental relationship due to direct and indirect effects: First, lacking legal recognition of the interparental relationship (through marriage or civil unions) may translate into a lack of pre-defined relational roles for same-sex relationships (e.g., Zamperini et al., 2016). While we found only scant evidence for this hypothesis in our review, this notion has also been put forward with regard to relationship uncertainty and ambiguity in a shifting sociopolitical climate for sexual minorities (Monk and Ogolsky, 2019). Conversely, legal recognition was found to strengthen the interparental relationship by publicly signaling love and commitment (e.g., Taylor, 2011; Kimport, 2013). However, legal recognition of the interparental relationship was frequently not found to be necessary for a loving and committed relationship (e.g., Vyncke and Julien, 2007; Goldberg and Kuvalanka, 2012; Kimport, 2013; DiGregorio, 2016).

Second, we found evidence within our review that the consequences of an unrecognized parent-child relationship might spill over into the interparental relationship due to a legal power differential between parents (e.g., Padavic and Butterfield, 2011; Butterfield and Padavic, 2014). Coupled with an unequal division of parenting tasks due to this legal power differential (e.g., Kazyak, 2015; Malmquist, 2015; Bacchus, 2018; Zhabenko, 2019), this can result in dependency, interparental conflict, and maladaptive counteractions (Padavic and Butterfield, 2011; Butterfield and Padavic, 2014). Conversely, evidence within our review suggests that legal recognition of the parent-child relationship prevents these consequences (Malmquist, 2015). Third, individual psychological strain due to legal vulnerability in the parent-child relationship (e.g., a worrying non-legal parent) can translate into strain on the interparental relationship, as evidenced in one study within our review (Butterfield and Padavic, 2014).

#### F.3: Coparenting

Drawing on the coparenting model (Feinberg, 2003), we propose that legal vulnerability impacts the way parents relate to each other in their child-rearing. Most evidently in our review, legal vulnerability impacted division of (parenting) labor, when the non-legal parent was unable to take on responsibilities that required a legal guardian (e.g., signing documents, taking the child to medical appointments, e.g., Surtees, 2011; Maxwell and Kelsey, 2014; Kazyak, 2015; Malmquist, 2015; Bacchus, 2018; Zhabenko, 2019, but see Polaškova, 2007). However, we propose that legal vulnerability may also impact other coparenting dimensions either directly or through interparental conflict (F.4). Further direct effects include instances where the legal parent takes over important parenting decisions (i.e., child-rearing agreement, e.g., Padavic and Butterfield, 2011), or undermines the parental role of the non-legal parent (i.e., support/undermining).

No study in our review investigated the impact of impaired coparenting due to legal vulnerability on child outcomes directly (F.5). However, the general association between coparenting and child outcomes is a well-established finding in the family literature for both mixed-sex (Teubert and Pinquart, 2010; McHale and Lindahl, 2011) and same-sex parent families (Farr and Patterson, 2013; Farr et al., 2019).

#### F.6: Parental Adjustment and Parenting

Important determinants of coparenting are individual parenting behaviors and aspects of parental adjustment, such as parental self-efficacy and parental mental health (Feinberg, 2003). Based on the evidence within our review (e.g., Surtees, 2011; Rawsthorne, 2013; Malmquist, 2015; Bacchus, 2018; Gash and Raiskin, 2018), we propose that legal vulnerability might impact parental self-efficacy of the non-legal parent in particular through feelings of parental illegitimacy, and legally determined constraints to engage in parenting (e.g., being able to take parental leave; Ross et al., 2005).

#### F.7: Parent-Child Relationship

Within the studies included in our review, the impact of legal vulnerability on the parent-child relationship was most noticeable in the case of relationship dissolutions (Goldberg and Allen, 2013; Malmquist et al., 2020), when the non-legal parent's means to gain custody for the child are limited or inexistent. Without informal agreements between parents (Goldberg and Allen, 2013), this was reported to result in a loss of contact to the child (similarly in case of death or incapacity of the legal parent)—a pervasive fear for non-legal parents (e.g., Hequembourg and Farrell, 1999; McClellan, 2001; DiGregorio, 2016; Bacchus, 2018). As described above, this fear can permeate family relationships even before a relationship dissolution, and, in some instances, lead to increased caution on the side of the non-legal parent with regard to the relationship with the child (McClellan, 2001; Padavic and Butterfield, 2011).

Conversely, the legal validation of the parent-child relationship (e.g., through a second-parent adoption) can serve to validate the relationship between parents and children, as evidence suggests (Goldberg et al., 2013, 2014; Gash and Raiskin, 2018). The joint efforts to mitigate legal disadvantages was also reported as strengthening the parent-child relationship (Gash and Raiskin, 2018). Some evidence also suggests that the legal recognition of the interparental relationship might strengthen the parental role of the non-legal parent (particularly in stepfamilies; Goldberg and Kuvalanka, 2012).

#### F.8: Sibling Relationship

Similar to the impact on the parent-child-relationship, legal vulnerability can affect the sibling relationship after a parental relationship dissolution. Specifically, siblings with different legal parents might be reared apart (see Goldberg and Allen, 2013; Malmquist et al., 2020, for evidence within our review).

#### F.9: Child Outcomes

Children might experience stressors related to the lacking legal recognition of family relationships or the criminalization of their parents' sexual orientation. Within our review, this included feelings of reduced family legitimacy (F.1), but also experiences of discrimination (Bos et al., 2008; Goldberg and Kuvalanka, 2012; Goldberg and Allen, 2013; Goldberg et al., 2013) or concealment of their family structure (Bos et al., 2008; Goldberg and Kuvalanka, 2012; Messina and D'Amore, 2018; Zhabenko, 2019). No study in our review tested the impact of experiencing these legal vulnerability-related stressors on child health. However, evidence on the impact of general minority-related stressors in children with same-sex parents bolsters this assumption (Gartrell et al., 2005; Bos and van Balen, 2008; Koh et al., 2019). Some evidence within our review suggests that legal vulnerability (or associated structural factors) might indeed pose a risk to children's well-being and adjustment directly (Bos et al., 2008; Goldberg and Kuvalanka, 2012; Lick et al., 2012; Goldberg et al., 2013).

Only one study in our review investigated the impact of sexual orientation laws on children's educational attainment using eight waves of data from the large-scale American Community Survey (Boertien and Bernardi, 2019). This study found no evidence of an association between state-wide marriage laws (or anti-discrimination legislation) on children's school progress in same-sex parent families. This is in line with meta-analytic (Fedewa et al., 2015), representative (e.g., Rosenfeld, 2010; Potter, 2012), and non-representative (e.g., Gartrell and Bos, 2010) evidence suggesting that (when controlling for important confounders such as family transitions or socioeconomic status) parental sexual orientation is not associated with adverse academic or cognitive outcomes for children (see Boertien and Bernardi, 2019, for a detailed methodological review).

The impact of legal vulnerability on parental outcomes (see above) may have indirect ramifications for child adjustment. Specifically, a plethora of studies established that impaired parental mental health (Goodman et al., 2011; van Santvoort et al., 2015), interparental conflict (Rhoades, 2008; Van Eldik et al., 2020), negative parent-child relationships (Erel and Burman, 1995; Popov and Ilesanmi, 2015), dysfunctional parenting (McLeod et al., 2007; Yap and Jorm, 2015), low parental self-efficacy (Albanese et al., 2019), and coparenting problems (Margolin et al., 2001; Teubert and Pinquart, 2010) belong to the primary family risk factors for child development. These indirect effects of legal vulnerability on child outcomes have yet to be tested empirically as no study in our review addressed them.

While not focal to our review, we also propose that the economic and legal disadvantages of unrecognized family relationships (e.g., lack of health insurance, Gonzales and Blewett, 2013) and, conversely, increased legal and financial security of the family (e.g., Goldberg and Kuvalanka, 2012; Gartrell et al., 2019; Malmquist et al., 2020) impact children's health and well-being. This robust association between parental socioeconomic status and child health is outlined in detail elsewhere (Repetti et al., 2002; Conger et al., 2010).

### Counteractions to Alleviate the Effects of Legal Vulnerability

Evidence for the delineated pathways in our model (particularly for family relationships) were not uniformly found within studies (see rightmost column of Table 3). This runs counter to minority stress and other stigma theories (Meyer, 2003; Hatzenbuehler, 2016). Adopting a systemic approach to family resilience (Walsh, 2016), we propose that same-sex parent families engage in counteractions (Figure 5) on the personal (C.1), family (C.2), and systemic level (C.3) to alleviate the impact of legal vulnerability. We do not make assumptions about the adaptivity of the counteractions presented therein. Similarly, some counteractions may serve several purposes and therefore may be placed within more than one level.

#### C.1: Within Person

Evidence within our review suggests that family members engage in person-centered counteractions such as emotion regulation (Butterfield and Padavic, 2014), seeking legal information (e.g., to protect their family or to regulate their emotions by looking for positive legal change in other countries; e.g., Dalton and Bielby, 2000; Short, 2007; Kazyak, 2015; Ollen and Goldberg, 2016), or questioning heteronormativity within legislation and family models (e.g., Hequembourg, 2004; Short, 2007; Rawsthorne, 2013; Zamperini et al., 2016; Vučković Juroš, 2019a).

#### C.2: Within Family

Family members engage in counteractions to mitigate legal vulnerability in other family members, as evidence within our review suggests. These relate to emotion regulation (e.g., parents instilling pride in their children about their family; e.g., Goldberg et al., 2013; Butterfield and Padavic, 2014; Maxwell and Kelsey, 2014; Ollen and Goldberg, 2016), emphasizing equal parenting (Goldberg and Allen, 2013; Malmquist et al., 2020), or creating legal dependency between family members (e.g., by obtaining second-parent adoption in the absence of legal partnership options; e.g., Hequembourg and Farrell, 1999; Dalton and Bielby, 2000; Acosta, 2017; Wheeler et al., 2018; Stambolis-Ruhstorfer and Descoutures, 2020).

Of note, three counteractions stem from only one study included in our pilot sample (Butterfield and Padavic, 2014). These counteractions are linked to the interparental relationship and focus on minimizing the probability of a relationship dissolution (which would entail loss of contact between the non-legal parent and the child). They entail the creation of emotional (e.g., by reducing other support systems or isolation of the partner) and financial dependency (e.g., by being the sole breadwinner), or acquiescing to the partner's wishes.

#### C.3: Within System

Family members engage in counteractions that are directed toward people or institutions outside the family unit. Within studies in our review, this entailed (legally) securing the family structure through obtaining wills or power of attorney (not surprisingly the most frequent counteraction; e.g., Dalton and Bielby, 2000; Bergen et al., 2006; Rostosky et al., 2016; Gash and Raiskin, 2018; Zhabenko, 2019), temporal or permanent relocation to gain legal security or recognition (e.g., Goldberg et al., 2013; Maxwell and Kelsey, 2014; Kazyak, 2015; Park et al., 2016) or safety (e.g., by seeking asylum; Zhabenko, 2019), or by concealing the family structure out of safety concerns (Zhabenko, 2019).

Evidence suggests that families also choose to ignore legal limitations (e.g., a non-legal parent acting as a legal guardian; e.g., Rawsthorne, 2013; Wheeler et al., 2018) or engage in symbolic actions such as commitment ceremonies or sharing the same last name (e.g., Bergen et al., 2006; DiGregorio, 2016; Zamperini et al., 2016; Bacchus, 2018; Wheeler et al., 2018). Families also advocate for legal change (Dalton and Bielby, 2000; Brown et al., 2009; Park et al., 2016; Gash and Raiskin, 2018; Wheeler et al., 2018), or actively engage other people in their family life (e.g., to create allyship or convey guardianship arrangements in the case of the legal parent's death; Dalton and Bielby, 2000; Butterfield and Padavic, 2014; Park et al., 2016; Gash and Raiskin, 2018).

#### Side-Effects and Reasons Not to Engage in Counteractions

None of the counteractions above provide the legal protection that would be automatically conferred by law and some may even have negative ramifications as our review suggests. Particularly strategies related to securing the family structure via other means were considered to be (too) costly, time-consuming, frustrating, or shameful (e.g., Rostosky et al., 2010; Denman, 2016; Bacchus, 2018; Gash and Raiskin, 2018). Other counteractions were described as inducing guilt, such as including a child in concealing the family structure (Messina and D'Amore, 2018), or creating financial or emotional dependency in a partner to prevent a relationship dissolution (Butterfield and Padavic, 2014).

### Moderators

#### M.1: Contextual Factors

First, our review identified important actors outside the family (see Figure 2) who can ignore or emphasize lacking legal ties between family members. By doing so, they alleviated or exacerbated legal vulnerability, for example judges and notaries concerned with second-parent adoptions or notarizing important documents (e.g., DiGregorio, 2016; Gash and Raiskin, 2018; Zhabenko, 2019), border control agents questioning the family structure (Gash and Raiskin, 2018; Vučković Juroš, 2019b), healthcare staff or teachers in their regard of non-legal parents (e.g., Polaškova, 2007; Surtees, 2011; Goldberg and Allen, 2013; Malmquist et al., 2020), or (un-)supportive families of origin (e.g., Hequembourg and Farrell, 1999; Goldberg et al., 2013; Vučković Juroš, 2019b).

Second, anecdotal evidence from other families experiencing legal disadvantages (e.g., Surtees, 2011; Goldberg and Kuvalanka, 2012; Butterfield and Padavic, 2014; Gash and Raiskin, 2018) or, conversely, lacking role models for same-sex parents (Sobočan, 2013) were found to exacerbate or mitigate the impact of lacking legal recognition, particularly with regard to safety concerns and worries. Due to lacking norms, rituals, and language for same-sex parent families in general (see e.g., Hall and Kitson, 2000), as well as an ambiguous legal climate (DiGregorio, 2016), we propose that these families might be particularly reliant on anecdotal evidence in navigating the legal system.

Third, the impact of legal recognition fluctuates in everyday family life. Within our review, it was found to be more salient while traveling (e.g., Gash and Raiskin, 2018; Vučković Juroš, 2019b), in a medical emergency (Bergen et al., 2006; Gash and Raiskin, 2018), or after a relationship dissolution (Hequembourg and Farrell, 1999; Goldberg and Allen, 2013; Malmquist et al., 2020). Thus, while legal vulnerability is defined as enduring, its influence varies and is exacerbated in situations where legal ties are (expected to be) relevant.

#### M.2: Family Characteristics

Characteristics of the family may moderate the impact of legal vulnerability or the family's ability to engage in counteractions. We found that different family types, such as planned (Polaškova, 2007), foster (Goldberg et al., 2013), or stepfamilies (Gash and Raiskin, 2018), may experience different ramifications of lacking legal recognition (e.g., stepfamilies with a second legal parent outside the family unit). Additionally, evidence suggests that family members may experience lacking legal recognition differently, for example a non-legal parent being impacted more directly by legal vulnerability (e.g., Padavic and Butterfield, 2011; Kazyak, 2015; Wheeler et al., 2018), or a child who is unknowing of the family's legal status (e.g., Ollen and Goldberg, 2016).

#### M.3: Couple Characteristics

Moderating characteristics on the couple level found in our review are the gender of the couple (e.g., Taylor, 2011; Goldberg et al., 2013), and their socioeconomic status (which enables the engagement in many counteractions, particularly those related to legally securing the family structure; e.g., Taylor, 2011; Goldberg and Kuvalanka, 2012; Kazyak, 2015; DiGregorio, 2016).

#### M.4: Individual Differences

Characteristics of the parents, including age (Reeves, 2011), personality traits (e.g., optimism, Ollen and Goldberg, 2016), minority stress experiences (Goldberg and Smith, 2011; Ollen and Goldberg, 2016), history of migration (Vučković Juroš, 2019b), past experiences with the legal system (McClellan, 2001; Butterfield and Padavic, 2014; Goldberg et al., 2014), as well as characteristics of the child, such as age (McClellan, 2001; Ollen and Goldberg, 2016) or gender (Bos et al., 2008; Goldberg and Kuvalanka, 2012), may moderate the impact of legal vulnerability or their ability to engage in counteractions, as our review suggests. Furthermore, the awareness about the current legal situation or the family's legal status may moderate their impact on family members (e.g., children being unaware about their non-legal relationship to one of their parents; Ollen and Goldberg, 2016; Malmquist et al., 2020).

With regard to minority stress in particular, one study included in our review found that parental internalized homonegativity moderated the association between a state's legal climate and changes in depression and anxiety during the 1st year of parenthood (Goldberg and Smith, 2011). On the individual level, minority stress has also been found to moderate the impact of sexual orientation laws (e.g., Bauermeister, 2014; Pachankis et al., 2014; Hylton et al., 2017; Ogolsky et al., 2019).

## Discussion

Based on a thematic synthesis of 55 studies, we introduced the legal vulnerability model for same-sex parent families that aims to link the impact of legal recognition of family relationships with minority stress and family theories on the individual, couple, and family level. We propose that legal vulnerability increases the risk for all family members of experiencing or expecting adverse outcomes in health- and family-related domains. Family members also actively engage in counteractions to alleviate the impact of legal vulnerability. Characteristics on the contextual, familial, couple, and individual level may moderate the impact of legal vulnerability or their ability to engage in counteractions.

Based on the evidence within our review, we assume that a legally secure family structure is in the best interest of all family members. The relatively scarce findings that suggest no or a counterintuitive impact of sexual orientation laws on certain outcomes (e.g., family relationships) should not be used as a justification for denying sexual (or other) minority groups' access to equal rights. Rather, they can be seen as evidence of the resilience that same-sex parent families show when maintaining loving and committed family relationships amidst an unfavorable legal climate and concurrent societal stigmatization.

### Strengths and Limitations of the Legal Vulnerability Model

A strength of our model is its empirical evidence base gathered via systematic literature search and synthesis. Its grounding in minority stress and family theories overcomes criticism in the field (Farr et al., 2017; van Eeden-Moorefield et al., 2018) and offers empirically testable pathways for future research and implications for clinical practice. By emphasizing the family unit (Minuchin, 1974), the model also moves away from the individuum-focused approach in minority stress research (LeBlanc et al., 2015). Our findings also suggest the need for a general family minority stress model that conceptualizes minority stress particular to parents and children.

Many of the proposed pathways in our model await empirical examination using rigorous, quantitative designs. Specifically, little is known about how legal vulnerability impacts family relationships and child outcomes and how it manifests itself in families deviating from the predominantly white, female-headed, and well-educated families within our sample (see below). Moreover, a concomitant examination of impact-related factors, moderating characteristics, and counteractions seems warranted to advance our understanding of legal vulnerability.

### Implications for Research and Practice

#### Operationalizing Legal Vulnerability: A Fruitful Challenge

Quantitative investigations of the pathways outlined in the legal vulnerability model are paramount considering the current primarily qualitative evidence base.

Across jurisdictions, we suggest including items related to family relationships, parenting, or child outcomes in multi-nation investigations into sexual minority health (e.g., European Union Agency for Fundamental Rights, 2020; Weatherburn et al., 2020). Research on sexual minority individuals has capitalized on the legal variation offered within these datasets by linking objective indices of the legal climate with health-related outcomes (Berg et al., 2013; Pachankis and Bränström, 2018; van der Star et al., 2021). This constitutes some of the most compelling evidence on the impact of sexual orientation laws (and concomitant societal attitudes) to date. Using legal variation across many jurisdictions (along with other country-level control variables) would overcome the limitations of the two country-comparisons found within our review (Vyncke and Julien, 2007; Bos et al., 2008; Shapiro et al., 2009).

Within jurisdictions, variation in legal vulnerability can be assessed in several ways. Many of the approaches outlined below have already been recommended in pertinent reviews on the advancement of research on same-sex couples and families in general (e.g., LeBlanc et al., 2015; Umberson et al., 2015) and can be extended to the structural level.

First, in jurisdictions without legally recognized relationships, dyadic designs (e.g., actor-partner interdependence models, Smith et al., 2020) can be used to contrast outcomes in legal and non-legal parents while taking further partner characteristics (e.g., minority stress) into account. Daily fluctuations in legal vulnerability and stress spillover effects could be assessed by dyadic diary studies (Totenhagen et al., 2018; Cooper et al., 2020).

Second, in jurisdictions with legally recognized relationships, investigations into “lingering” or continued effects of legal vulnerability (e.g., on parental legitimacy or legal rejection sensitivity) seem warranted. Legal advances for sexual minorities are recent phenomena globally and same-sex parent families today are likely to have faced legal vulnerability at some point in their shared family biography in the past. Indeed, referenda and campaigns on sexual minority rights can exert long-lasting influences on sexual minorities (Russell et al., 2011). Lifeline and relationship timeline approaches (LeBlanc et al., 2015) could elucidate lingering effects of legal vulnerability. Research with adult children would provide insights into lingering effects of legal vulnerability beyond childhood (see e.g., Lick et al., 2012).

Third, researchers could draw on legal variation within a jurisdiction by assessing outcomes before and after legal change (i.e., akin to a quasi-experimental design; see Hatzenbuehler et al., 2009, 2010). This entails re-analyzing existing datasets or capitalizing on periods of legal changes as they take place.

The proposed research designs necessitate psychometric innovations to measure the legal climate for same-sex parent families and aspects of legal vulnerability on the individual level. On the country level, several indices to measure the general (socio-)legal climate for sexual minorities have been recently developed (e.g., Lee and Ostergard, 2017; Lamontagne et al., 2018) that could be adapted to assessing the legal climate for same-sex parent families in particular.

On the individual level, we suggest developing and validating measures that capture different manifestations of legal vulnerability (e.g., legal worries, family legitimacy due to [lacking] legal recognition) and family members' counteractions to tackle legal vulnerability. Researchers can utilize existing scales that measure aspects of legal vulnerability directly (e.g., legal worries; Shapiro et al., 2009). They can also adapt existing scales, such as couple-level minority stress (Neilands et al., 2020), parenting stigma (Gato et al., 2019; Shenkman, 2020), or challenges in achieving parenthood (Simon and Farr, 2020) to explicitly incorporate legal aspects.

#### Investigating Legal Vulnerability: Areas for Future Research

The development of models related to sexual minority health on the individual, couple, and family-level has proliferated in recent years. Future research could integrate legal vulnerability in the family resilience model (Prendergast and MacPhee, 2018), the relationship uncertainty model (Monk and Ogolsky, 2019), or adaptations of the vulnerability-stress-adaptation model (Karney and Bradbury, 1995; Totenhagen et al., 2018), among others. Conversely, researchers could link other concepts of legal meaning making for sexual minorities, such as legal consciousness (Hull, 2016), with legal vulnerability. Furthermore, assuming a rather contrasting theoretical perspective to minority stress (Meyer, 2003) and family systems theory (Minuchin, 1974)—for example through a psychoanalytical lens—could help elucidate and refine the epistemological boundaries of the legal vulnerability model and ultimately strengthen its value for theory and practice. Methodologically, a more interpretative approach to research synthesis—for example grounded theory synthesis (Eaves, 2001) or critical interpretative synthesis (Dixon-Woods et al., 2006)—could prove fruitful in analyzing the discursive strategies that study participants used.

Comparatively little is known about how legal vulnerability impacts family functioning, relationships, and child outcomes. Our evidence suggests that children are relatively unaffected by legal vulnerability (as compared to their parents), particularly in their family relationships and functioning (Malmquist et al., 2020), although reporting biases or normalizing strategies cannot be ruled out (Clarke and Demetriou, 2016). This adds to the robust body of evidence that documents how children with same-sex parents fare well-even in stigmatizing environments (see Pollitt et al., 2020, for a review).

The degree of spillover of legal vulnerability into family life can be considered the key determinant of how far it impairs the child's well-being. Concurrent assessments of parents and children could shed light on the degree to which same-sex parents are able to “compartmentalize” legal vulnerability and avoid a spillover into the parent-child relationship or parenting practices (consequently influencing child outcomes).

With regard to populations, our sample is biased toward white, well-educated (presumably), cisgender, and female-headed families. Future research should strive for including diverse families in terms of parental gender and sexual orientation (e.g., bisexual parents in same-sex relationships, unique legal vulnerabilities of transgender parents), race/ethnicity, and socioeconomic background, while incorporating an intersectional approach (Bowleg, 2012) that is sensitive to the complex effects of multiple and intersecting discrimination.

#### Working With Legal Vulnerability: Implications for Clinical Practice

First, legal vulnerability for same-sex parent families is caused and perpetuated by the lacking legal recognition of family relationships, just as minority-specific drivers of sexual minority health disparities are caused and perpetuated by structural and societal stigmatization (Pachankis, 2015). In line with recommendations made by the American Psychological Association (2020a,b), clinical practitioners should promote the beneficial effects of protective legislation as being in the best interest of their clients.

Second, legal vulnerability needs to be acknowledged as a source of psychological strain for all family members in clinical practice. This entails adapting mixed-sex family therapy programs and minority stress related approaches for individuals or couples (Pachankis, 2018; Burton et al., 2019; Pepping et al., 2020) to the needs of legally vulnerable families.

Our model bears several implications for the derivation of such programs: Clinicians can use the counteractions proposed in our review to delineate counseling approaches for legally vulnerable same-sex parent families. Many counteractions found within our review map onto well-established family resilience processes (Walsh, 2003, 2016), such as meaning making of adversity (e.g., seeking legal information, overcoming heteronormativity), cooperative parenting, family connectedness, and mobilizing social and economic resources. Moreover, counteractions related to legal vulnerability have been identified as buffering against individual-level minority stress or promoting well-being in sexual minorities and their relationships (see e.g., Kwon, 2013; Hill and Gunderson, 2015): These include managing disclosure (Oswald, 2002; Kwon, 2013), activism (Oswald, 2002; DeBlaere et al., 2014), symbolic rituals and naming practices (Oswald, 2002), choosing kin, reframing minority stress experiences (Oswald, 2002; Frost, 2014), supportive dyadic coping (Randall et al., 2017), instilling resilience in children (Oakley et al., 2017), and legally securing the family structure (Oswald, 2002; Riggle et al., 2005).

Our list of moderators might be used to identify families (or individuals) that are particularly vulnerable or, conversely, equipped with many resources (e.g., families with a high socioeconomic status). Resilience-focused therapy and counseling approaches could aim at deriving useful coping strategies for dealing with legal vulnerability as parents and outlining effective and age-appropriate strategies to address legal vulnerability in children. However, the integration of legal vulnerability into clinical practice is by no means limited to structured therapy programs. For example, informal meeting groups have been found to be an important source of social support but also legal information for same-sex parents (Kazyak, 2015; Álvarez-Bernardo and García-Berbén, 2018; Appelgren Engström et al., 2019). Group facilitators (e.g., community members or clinicians) serve as important multiplicators in communicating the potential impact of legal vulnerability on these families and should be knowledgeable about adaptive counteractions in particular.

In jurisdictions with legal recognition for same-sex parent families, we argue that it is important to address possible lingering effects of legal vulnerability. For example, a study on same-sex couples (post marriage legislation) found that perceived unequal relationship recognition (e.g., feelings of the relationship being treated as “second-class” by the government) predicted adverse mental health outcomes irrespective of legal relationship status (LeBlanc et al., 2018). Clinicians should be prepared to address relics of past legal vulnerability on the individual (e.g., maladaptive beliefs about parental legitimacy) or family level (e.g., perceived unequal relationship recognition).

Clinicians should also be mindful about the legal shifts that have characterized the past decade in many countries, but they should not be oblivious to more insidious forms of minority stress that continue to shape sexual minorities' lives. Experiencing minority stress does not end when legal equality sets in, as evidence from our review (e.g., Rawsthorne, 2013; Goldberg et al., 2014; Gash and Raiskin, 2018) and elsewhere (Riggle et al., 2018; Wootton et al., 2019; European Union Agency for Fundamental Rights, 2020) suggests.

### Limitations

First, we excluded studies that (i) focused on the impact of sexual orientation laws on family formation, (ii) were concerned with same-sex couples with unclear parental status, (iii) investigated discrimination unrelated to the legal system, or (iv) investigated family constellations with one or more than two parents. All of these studies are beyond the scope of our review and necessitate their own synthesis. Still, some of these studies may have yielded responses by participants that would have warranted inclusion in our review (e.g., studies concerned with family formation that reported data on the 1st years of parenthood).

Second, we did not systematically search for gray literature excepting our forward-backward search. Publication and other dissemination biases are typically framed as a threat to the synthesis of quantitative studies (Rothstein et al., 2005) but they can also impair qualitative evidence syntheses (Petticrew et al., 2008; Toews et al., 2017). However, the well-known schism between deficit- and resilience-oriented approaches in the study of sexual minority health (Frost, 2017; Prendergast and MacPhee, 2018) makes the direction of this bias hard to predict: Researchers could be inclined to draw attention to the detrimental impact of lacking legal recognition, but they could also be interested in emphasizing the resilience of same-sex parent families in a legally unfavorable climate. Because our review aimed to propose a framework (thus aiming for saturation of themes), we do not regard a possible impact of publication bias as a threat to the validity of our results. Still, future research would benefit from systematically searching various gray literature databases and sources from human rights organizations to look for convergence with the themes identified in our review.

In a similar vein, we only included studies published in English or German. This language bias might have resulted in the omission of studies investigating the impact of criminalizing laws in particular, as they might have been published in their native language or in a non-traditional outlet not to be found via our database search. Furthermore, our sample is heavily biased toward Western countries (with almost two thirds of studies originating from the US in particular) and only includes one study (Zhabenko, 2019) concerned with criminalizing legislation.

Third, we did not systematically stratify our results by different laws. We did not deem this distinction useful, as same-sex parent families often reported on the impact of lacking legal recognition of both the interparental and parent-child relationship. Future research might benefit from delineating the impact of specific laws (e.g., marriage) on key outcomes.

Fourth, synthesizing results across studies from countries with varying sociolegal climates gives rise to a possible decontextualization of findings (Thomas and Harden, 2008). The legal context must be regarded as part of a larger political context that shapes the experiences of same-sex parent families. For example, countries with the same level of legal recognition might differ with regard to how this legal recognition came about (e.g., via a court decision, a referendum, or a parliament vote) and how it is perceived by society. Our study's aim (i.e., the postulation of a valid model across different studies and contexts) required this decontextualization to some degree. Still, future research should take into account a country's surrounding sociopolitical climate (termed structural stigma, Hatzenbuehler and Link, 2014, in our model) when investigating legal vulnerability.

Fifth, the conflation of societal prejudice (arguably higher in countries with lacking legal recognition, Smith et al., 2014), lacking biological ties to a child, and lacking legal ties (to a child or a parent) in qualitative reports posed a challenge during the coding process. Our conservative coding strategy strengthens the validity of our findings with regard to legal vulnerability, but undoubtedly fails to capture the lived experiences of same-sex parent families in a society that is characterized by both legal and societal prejudice against them.

### Conclusion

In this systematic review, we introduced the novel concept of legal vulnerability that serves to link sexual orientation laws impacting same-sex parent families with parental, child, and family outcomes. Many of the complex and reciprocal pathways outlined in our model have yet to be put to rigorous empirical tests. Yet, it is not premature to claim that a legally secure family structure is not only from a human rights perspective, but also from a psychological perspective in the best interest of both parents and children.

## Data Availability Statement

The datasets presented in this study can be found in online repositories. The names of the repository/repositories and accession number(s) can be found at: https://osf.io/qs6hp/.

## Author Contributions

MS, CA, NM, and MZ conception, design of study, analysis, interpretation of data, and approval of final version. MS, CA, and NM acquisition of data. MS drafting the manuscript. CA, NM, and MZ revised the manuscript critically for important intellectual content. All authors contributed to the article and approved the submitted version.

## Conflict of Interest

The authors declare that the research was conducted in the absence of any commercial or financial relationships that could be construed as a potential conflict of interest.

## Footnotes

^1^Importantly, sexual orientation laws also regulate the access to reproductive technologies. However, the impact of legislation on family formation in same-sex parent families is beyond the scope of this review.

^2^Sexual minorities are impacted by the legislation in their country, but also by concomitant societal attitudes (Hatzenbuehler et al., 2017). The impact of societal attitudes and compound effects of laws and attitudes (collectively referred to as “structural stigma”, Hatzenbuehler and Link, 2014) are depicted in our model for the sake of completeness, but not discussed in detail.

^3^Notably, various other family forms may face legal vulnerability, including immigrant families (Brabeck et al., 2016) or stepfamilies (Cherlin, 1978), but these are outside the scope of this review.
